# Automated *para*-Hydrogen Hyperpolarization for Mixture Analysis Using ^1^H and ^13^C Benchtop NMR Detection

**DOI:** 10.1021/acs.analchem.6c01237

**Published:** 2026-06-20

**Authors:** Daniel A. Taylor, James McCall, Fraser Hill-Casey, Jonathan Hedges, Izzy Hehir, Gregory J. Yule, Stuart Murray, Abigail Mortimer, Meghan E. Halse

**Affiliations:** † Department of Chemistry, 8748 University of York, Heslington, York, North Yorkshire YO10 5DD, U.K.; ‡ Research IT, University of York, Heslington, York, North Yorkshire YO10 5DD, U.K.

## Abstract

Benchtop NMR spectroscopy is an affordable and accessible technique for mixture analysis but suffers from lower sensitivity and increased signal overlap compared to standard high-field NMR. Signal amplification by reversible exchange (SABRE) hyperpolarization can effectively address the sensitivity limitation, with automation of the hyperpolarization step enabling the multistep NMR experiments required to resolve signal overlap for complex mixture analysis. The automated workflow presented herein delivers highly repeatable hyperpolarization (with a 2.5% relative standard deviation in signal enhancement across 100 experiments) and can be integrated into any NMR pulse sequence with typical repolarization times of ∼13 s. Using this approach, a fully resolved ^13^C­{^1^H} benchtop NMR spectrum with signal-to-noise ratios of up to 40 for a mixture of pyridine, 4-methylpyridine, and 3,5-dimethylpyridine (3 mM each) at natural isotopic abundance is obtained with only 18 min of signal averaging. Overlapped peaks in the ^1^H NMR spectrum of this mixture are resolved using a SABRE-enhanced 2D ^13^C–^1^H HETCOR experiment (8 scans, 2.4 h). Analysis of a lower concentration mixture (750 μM per analyte) is exemplified with a SABRE-enhanced 2D ^1^H–^1^H COSY spectrum acquired in a single scan in 37 min.

## Graphical abstract

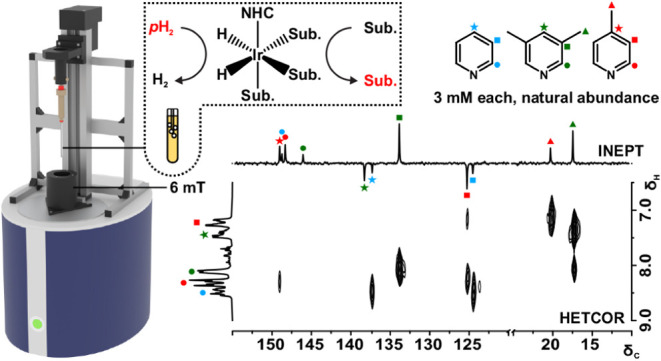

## Introduction

Benchtop NMR offers a more accessible alternative to conventional high-field NMR for probing liquid-state molecular structure and dynamics and is gaining popularity for applications such as the study of reaction kinetics,^1–3^ detection of food or pharmaceutical adulteration,^4–8^ and monitoring of batch processes.^9–11^ However, when compared to their high-field (*B*_0_ ≥ 7 *T*) counterparts, the moderate magnetic field strengths (1 < *B*_0_ < 2.1 *T*) of current permanent magnet-based benchtop NMR spectrometers result in lower sensitivity and increased peak overlap. Spectral congestion, which is particularly acute in the context of mixture analysis using ^1^H NMR, can in some cases be addressed through the detection of NMR-active nuclei with high receptivity and broad chemical shift ranges, such as ^31^P^12,13^ and ^19^F.^14,15^ ^13^C also benefits from a broad chemical shift range, but its low receptivity generally makes its detection on benchtop NMR spectrometers impractical without isotopic enrichment.

A proven approach to increase the sensitivity of benchtop NMR spectroscopy is through hyperpolarization,^16^ where a large non-Boltzmann nuclear spin state population distribution is created to transiently amplify the measured NMR signals. Several hyperpolarization approaches, including Overhauser,^17–21^ dissolution dynamic nuclear polarization (DNP),^22–27^ photochemically induced dynamic nuclear polarization (photo-CIDNP),^28,29^ and *para*-hydrogen-induced polarization (PHIP),^30–37^ have been integrated with benchtop NMR detection. PHIP is particularly promising for its simple instrumentation and its ability to generate high levels of hyperpolarization in seconds, without compromising the accessibility or throughput of benchtop NMR spectroscopy.

In PHIP, the nuclear singlet state of molecular hydrogen, *para*-hydrogen (*p*-H_2_), is the source of polarization.^38^ A symmetry-breaking chemical reaction transforms this NMR-silent singlet state into observable hyperpolarization of a target molecule, either through irreversible hydrogenation of an unsaturated precursor^39–42^ or, in the signal amplification by reversible exchange (SABRE) approach,^43^ via reversible coordination of both the molecule and *p*-H_2_ at a catalytic center.^44^ In SABRE, the exchange of both *p*-H_2_ and the target molecule with the polarization transfer catalyst (Figure 1) promotes the buildup of hyperpolarization in free solution and allows repeated hyperpolarization of the same target molecule, as long as fresh *p*-H_2_ is supplied.

**Figure 1:**
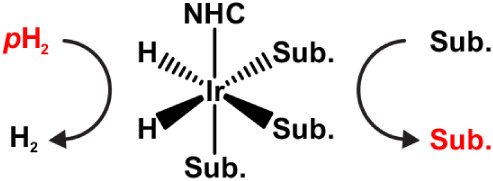
Schematic of the reversible exchange reaction that drives SABRE hyperpolarization, where the N-heterocyclic carbene (NHC) used in this work is 1,3-bis­(2,4,6-trimethylphenyl)­imidazol-2-ylidene (IMes), and Sub is the molecule to be hyperpolarized.

Polarization transfer in SABRE occurs through the transient scalar coupling network that exists between the *p*-H_2_-derived hydride ligands and the target molecule in the active SABRE catalyst. This transfer is field-dependent.^45,46^ For hyperpolarization of ^1^H, a polarization transfer field (*B*_PTF_) of ∼6.5 mT is effective for a wide range of analytes.^47^ Direct transfer to heteronuclei usually requires *B*_PTF_ values in the nT to μT range.^48^ Heteronuclei such as ^13^C or ^19^F can also be hyperpolarized through indirect transfer from ^1^H, either spontaneously through the formation of multispin correlations at *B*_PTF_ ≈ 6.5 mT,^30,49^ or by using pulse sequences such as INEPT (Insensitive Nuclei Enhanced by Polarization Transfer) at the NMR detection field.^50–52^ The required *B*_PTF_ is easily accessed using a permanent magnet Halbach array^53^ or solenoid,^30^ shielded from the Earth’s magnetic field where necessary. Integration of SABRE with benchtop NMR detection is straightforward, but the need to transfer the sample between *B*_PTF_ and the NMR detector presents a challenge for achieving the repeatable levels of hyperpolarization required for enhancing multistep NMR experiments.

A number of RF-driven polarization transfer schemes have been introduced that generate hyperpolarization within the NMR detection field.^54–61^ These *in situ* approaches remove the need to transfer the sample, enabling multistep experiments such as signal averaging. However, RF transfer is usually selective, and therefore multiple experiments are required to enhance resonances across the entire spectrum.^62^ Alternatively, the broadband hyperpolarization of standard SABRE can be preserved by automating the sample transfer step using flow,^30,63–65^ or by mechanical transfer employing either a robotic arm^66–68^ or a motorized linear actuator.^69,70^ Mechanical approaches typically provide faster transfer and, therefore, shorter repolarization times between each step of an NMR experiment.

In previous work, we used a stopped-flow system to combine SABRE hyperpolarization with 2D ^1^H–^1^H correlation spectroscopy (COSY)^30^ and ^1^H–^13^C heteronuclear correlation (HETCOR)^52^ benchtop NMR experiments. This flow-based approach was limited by long repolarization times (*ca*. 1 min) and solvent evaporation. Here, we describe an improved automated SABRE workflow consisting of a *p*-H_2_ converter, solenoid valve manifold, and linear actuator that overcomes these limitations. Off-the-shelf microcontrollers and simple macros embedded into the benchtop NMR spectrometer software are used to control *p*-H_2_ mixing and sample shuttling. The efficiency and repeatability of the hyperpolarization are analyzed and compared to manual sample transfer. SABRE-enhanced 1D ^13^C­{^1^H} benchtop NMR is applied to the analysis of mixtures at natural abundance, exploiting signal averaging to reduce the limit of detection. Finally, we demonstrate SABRE-enhanced 2D ^1^H–^13^C HETCOR and ^1^H–^1^H COSY benchtop NMR experiments, where repeatable generation of SABRE hyperpolarization enables the sampling of multiple dimensions to facilitate spectral assignment.

## Methods

### Sample Preparation Details

Pyridine, 4-methylpyridine, 3,5-dimethylpyridine, and methanol were purchased from Thermo Fisher Scientific. 3,5-Difluoropyridine was purchased from Fluorochem. Methanol-*d*_4_ was purchased from Sigma-Aldrich. All chemicals were used as received, without further purification. The SABRE precatalyst [IrCl­(COD)­(IMes)] was synthesized in-house according to a literature procedure.^71^ A range of procedures for the laboratory-scale synthesis of this commonly used precatalyst is available.^72–77^

The sample used for repeatability studies (Figure 4) contained 100 mM 4-methylpyridine and 5 mM [IrCl­(COD)­(IMes)] SABRE precatalyst in methanol. 19.5 μL of 4-methylpyridine and 6.4 mg of [IrCl­(COD)­(IMes)] were added to 1980.5 μL of methanol. The sample was sonicated for 10 min to aid dissolution.

The sample used for 1D ^13^C­{^1^H} signal-averaged experiments (Figure 5) and 2D ^13^C–^1^H correlation experiments (Figure 6a) contained 3 mM each of pyridine, 4-methylpyridine, and 3,5-dimethylpyridine, and 600 μM [IrCl­(COD)­(IMes)] SABRE precatalyst in methanol. 4.03 μL of pyridine, 4.87 μL of 4-methylpyridine, 5.68 μL of 3,5-dimethylpyridine, and 6.4 mg of [IrCl­(COD)­(IMes)] were added to 1985.40 μL of methanol. A 120 μL aliquot of this solution was subsequently added to 880 μL of methanol. The sample was sonicated for 10 min to aid dissolution.

The sample used for 2D ^1^H–^1^H correlation experiments (Figure 6b) contained 750 μM each of pyridine, 4-methylpyridine, 3,5-dimethylpyridine, and 3,5-difluoropyridine, along with 25 mM dimethyl sulfoxide (DMSO) and 5 mM [IrCl­(COD)­(IMes)] SABRE precatalyst in methanol-*d*_4_. Specifically, 16.11 μL of pyridine, 19.46 μL of 4-methylpyridine, 22.70 μL of 3,5-dimethylpyridine, and 18.33 μL of 3,5-difluoropyridine were added to 1923.40 μL of methanol-*d*_4_. A 7.50 μL aliquot of this solution, 1.78 μL of DMSO and 6.4 mg of [IrCl­(COD)­(IMes)] were subsequently added to 990.72 μL of methanol-*d*_4_. The sample was sonicated for 10 min to aid dissolution.

### Automated SABRE Apparatus

Achieving repeatable SABRE hyperpolarization with an automated workflow requires a means of mixing *p*-H_2_ into the solution while exposing the sample to the desired *B*_PTF_, followed by the rapid transfer of the sample into the NMR spectrometer for detection. All steps need to be carefully synchronized to ensure consistent and repeatable results both within and between experiments, and so they should be software-controlled. Here, we achieve this using the workflows outlined in Figure 2 and the sample shuttling apparatus presented in Figure 3. Full technical details, schematics, and bills of materials are available in the Supporting Information.

**Figure 2:**
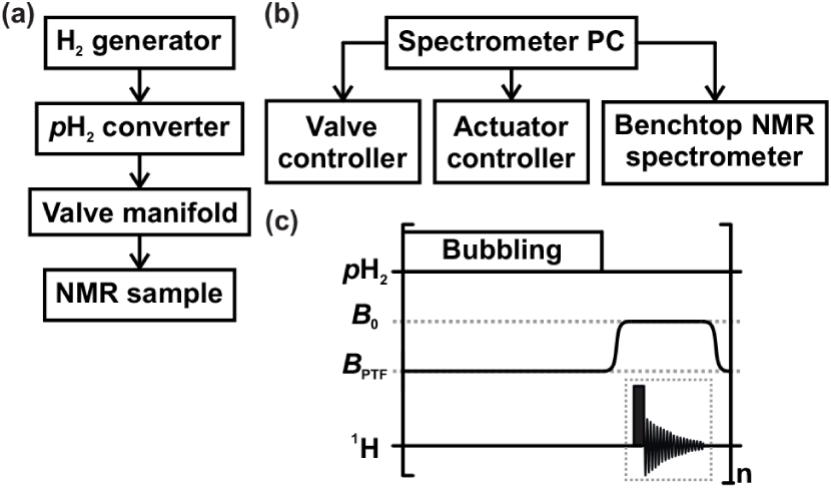
Block diagrams illustrating (**a**) the hardware components used for *p*-H_2_ generation and handling, and (**b**) the control architecture for *p*-H_2_ mixing, sample shuttling, and NMR detection. (**c**) Schematic pulse sequence for automated SABRE hyperpolarization. At the start of an experiment, the actuator positions the sample within the Halbach array (*B*_PTF_) and the valve controller initiates *p*-H_2_ bubbling for the desired polarization transfer delay (typically 10 s). Next, the sample is shuttled to the detection region (*B*_0_), and following a short settling delay (typically 230 ms), the pulse sequence is executed. In multistep experiments, the sample returns to the *B*_PTF_ after acquisition to repeat the cycle.

**Figure 3:**
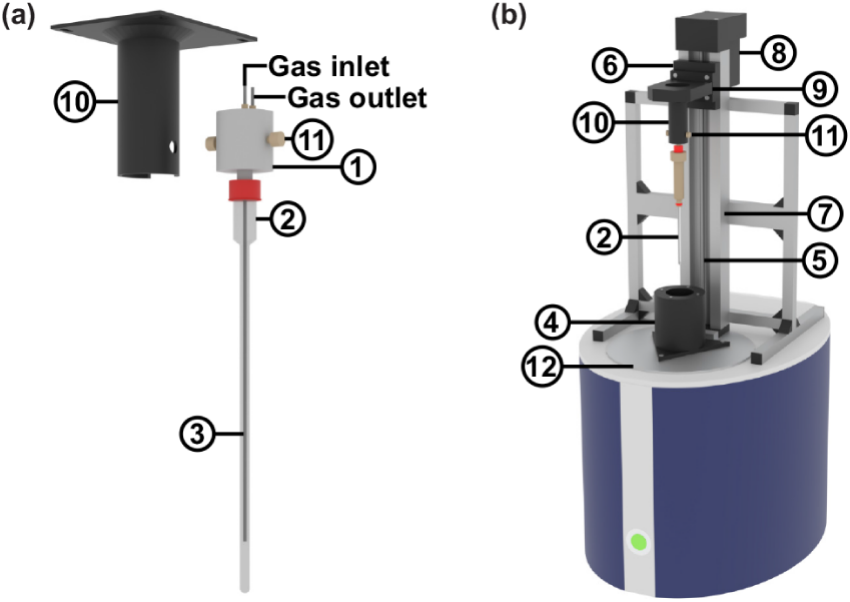
3D renders of the automated SABRE experimental setup. (**a**) The gas adaptor (**1**), NMR tube (**2**), and holder (**10**) used to interface the NMR sample with the linear actuator. The adaptor threads onto the NMR tube and contains ports for *p*-H_2_ delivery/return via 1/16″ capillary tubing (**3**) terminated above the detection region. The adaptor sits within the holder, secured by two screws (**11**). Gas lines are routed through the holder’s central bore. (**b**) The sample shuttling apparatus is installed on a Magritek Spinsolve benchtop NMR spectrometer using a machined base plate (**12**). The holder (**10**) attaches to an aluminum bracket (**9**) bolted to the actuator’s gantry plate (**6**). A motor (**8**) drives a lead screw (**5**) to move the gantry along the linear rail (**7**), shuttling the sample between the polarization transfer (*B*_PTF_) and NMR detection (*B*_0_) fields. A permanent magnet Halbach array (**4**) provides the *B*_PTF_ = 6.39 ± 0.14 mT.

A commercial hydrogen generator produces H_2_ with a maximum flow rate of 640 mL·min^–1^ at up to 10.5 bar of pressure. This is enriched to 51% *p*-H_2_ using a liquid nitrogen-cooled *p*-H_2_ converter, similar to others described in the literature.^78,79^ The delivery of *p*-H_2_ to the sample is controlled by a series of solenoid isolation valves, capable of gating gas flow within 30 ms. Combined with upstream pneumatic regulation and downstream mass flow control, the solenoid valve manifold ensures precise and consistent *p*-H_2_ flow through the sample at a maximum pressure of 5.5 bar (absolute). This pressure limit is set to ensure safe operation within the pressure rating of the glassware used in the system. To counter the effects of solvent evaporation over multiple experiments, the *p*-H_2_ passes through a 2 mL solvent reservoir included in-line before the sample tube.

The valve manifold interfaces with the sample tube through a custom two-part adaptor (**1**), which screws onto a 5 mm O.D. NMR tube sealed to a GL14 thread (**2**) (Figure 3a). This adaptor features central and offset threaded ports (suitable for IDEX Super Flangeless fittings) to provide an inlet for *p*-H_2_ delivery and an outlet for the return gas flow. Although more complex to implement than other methods in the literature (e.g., gluing capillaries to the cap of an NMR tube),^69^ the machined adaptor provides a robust, gastight connection that remains reliable over thousands of experiments. The assembly is compact and isolable, making it suitable for use with air-sensitive samples prepared in a glovebox. It is also quickly disassembled to simplify cleaning and maximize sample throughput.

Gas is delivered to the sample via 1/16″ O.D. capillary tubing (**3**) that is terminated above the detection region. This positioning is suboptimal for *p*-H_2_ dissolution but, in our practical experience, is an essential compromise when observing ^1^H nuclei on a benchtop NMR spectrometer, as the narrow chemical shift range makes spectral quality acutely sensitive to field distortions. The direction of *B*_0_ in benchtop NMR spectrometers is typically perpendicular to the bore axis. Introducing a capillary into the detection region, therefore, causes significant field inhomogeneity and broadening of the lines. For heteronuclear-detected experiments, where sensitivity is more important and spectral resolution and peak overlap are less of a concern, a narrow capillary can be extended along the entire length of the sample to improve gas mixing, as demonstrated by Barskiy and coworkers.^62,67^

To generate ^1^H SABRE hyperpolarization, the exchange reaction with *p*-H_2_ is carried out in a *B*_PTF_ = 6.39 ± 0.14 mT, generated by a permanent magnet Halbach array (**4**) situated on top of the benchtop NMR spectrometer (Figure 3b). The Halbach array is based on a previously published design,^53^ with the length and inner dimensions optimized to accommodate the NMR sample during transfer.

Following the generation of hyperpolarization at *B*_PTF_, the sample is transferred into the NMR spectrometer for detection using a commercially available linear actuator (Figure 3b). It consists of an 8 mm lead screw (**5**) coupled to a gantry plate (**6**), providing 400 mm of travel along a 500 mm linear rail (**7**). A stepper motor (**8**) synchronously drives the lead screw 1:1, with a linear velocity of 260 mm·s^–1^ and an acceleration of 930 mm·s^–2^ for a total sample transfer time of 1.27 s. The actuator interfaces with the NMR sample using an aluminum bracket (**9**) bolted to the gantry plate, to which a 3D-printed holder (**10**) is attached. Two screws (**11**) securely hold the gas adaptor (**1**) in place, ensuring the sample tube (**2**) remains parallel to the bore of the NMR spectrometer while in motion. A machined aluminum base plate (**12**) mounts the actuator on top of the Magritek Spinsolve benchtop NMR spectrometer using three screws, which precisely align the sample tube concentrically with the bore. Installation of the actuator requires no modification of the NMR spectrometer, other than the removal of the factory-supplied top plate. Only modifications to the aluminum base plate would be required to install the actuator on benchtop NMR instruments from other manufacturers.

The solenoid valve manifold and sample actuator are driven by programmable microcontrollers, which interface with the spectrometer control PC via separate serial connections. Graphical user interfaces (GUIs) developed within the SpinsolveExpert software (Magritek Limited) permit direct user operation of the valve manifold and actuator for system setup. For routine data collection, control commands are integrated directly into the NMR experiment. This ensures that *p*-H_2_ delivery and sample transfer are executed automatically prior to the pulse sequence. Importantly, these commands can be embedded into any existing NMR experiment within the SpinsolveExpert software environment or those developed by the user. SABRE hyperpolarization is refreshed between each step of the NMR experiment, as illustrated in the pulse sequence in Figure 2c, where any pulse sequence can be implemented within the dashed box. The total time to refresh the SABRE hyperpolarization between acquisition steps is typically ∼13 s, using a polarization transfer time of 10 s. Software for the GUIs and example pulse sequence software with embedded *p*-H_2_ bubbling and sample shuttling commands are provided in the data repository associated with this publication.

Several features are incorporated into the automated workflow to ensure operator and instrument safety. Digital sensors monitor gas pressure throughout the system, featuring a two-color display to alert users of pressure deviations indicative of a gas leak. An array of status LEDs provides additional visual feedback on the state of the solenoid valves. The *p*-H_2_ converter is protected by a proportional relief valve to safely vent excess pressure in the event of coil warm-up, while the integrated hydrogen generator automatically stops hydrogen production upon detection of a leak. To prevent sporadic movement, the output current of the actuator’s stepper motor defaults to 0% when not actively engaged in an experiment. An initialization sequence calibrates the actuator’s position at the start of every experiment. Software limits prevent out-of-range movement of the actuator, and optical limit switches are installed at the upper and lower limits of travel as a hardware fail-safe.

## Results and Discussion

### Repeatability of SABRE Hyperpolarization

The efficacy of the automated SABRE workflow is illustrated by the ^1^H benchtop NMR spectra of 100 mM 4-methylpyridine, with and without SABRE hyperpolarization, in Figure 4a. All three resonances are significantly enhanced without any loss of spectral resolution. To explore the benefits of automating the hyperpolarization process, one hundred repeat ^1^H pulse-acquire experiments were performed with the automated SABRE workflow and compared to manual transfer. In the manual approach, the sample is prepared in an NMR tube fitted with a J Young valve. Prior to each acquisition, the headspace of the NMR tube is evacuated and then charged with fresh *p*-H_2_. The sample is shaken at *B*_PTF_ for several (∼10) seconds before it is manually transferred (∼2 s) to the NMR spectrometer for detection.

Figure 4b presents the resultant enhancement factors for each of the three resonances of 4-methylpyridine. While both approaches produce comparable enhancement of all three ^1^H NMR signals, the manual method delivers poor repeatability across the series (5.0%, 5.9%, and 6.7% relative standard deviation for the *ortho*, *meta*, and methyl signal enhancement factors, respectively), even when performed sequentially by an experienced operator. This dispersion arises primarily from inconsistent *p*-H_2_ mixing during sample shaking and variable sample transfer times, both intrinsic sources of random error that cannot be entirely eliminated by user experience. The difference in relative standard deviation values is consistent with the differential longitudinal relaxation rates of the nuclei; the methyl protons, possessing the shortest *T*_1_ time constant, are disproportionately sensitive to inconsistency in the transfer duration.

The automated workflow provides at least a 2-fold improvement in repeatability, reducing the relative standard deviation for all three signal enhancement factors to 2.5%. This is an improvement over our previous flow-based system (*ca*. 5%)^30^ and is comparable to other mechanical transfer systems.^69,70^ Importantly, the improvement in repeatability comes at no detriment to signal enhancement; the larger *p*-H_2_ pressure differential (5.5 bar vs 4.0 bar absolute in manual experiments), combined with faster sample transfer, effectively compensates for the reduced mixing efficiency of gas bubbling. The consistent standard deviation across the three resonances indicates that the *T*_1_-dependent variation in the enhancement factor, caused by irregular sample transfer durations, has been eliminated. The residual scatter can be attributed to random variations in the gas–liquid mixing efficiency. This hypothesis is further supported by the fact that the scatter in enhancement factors is highly correlated between the three resonances. The ratio of enhancement factors for the *ortho* and *meta* resonances has a relative standard deviation of only 0.3% across the 100 experiments. This suggests that it is the overall hyperpolarization available (i.e., the concentration of *p*-H_2_ in solution) that varies between repeat experiments, and not any of the other factors affecting the efficiency of polarization transfer, such as the field profile during transfer or the transfer time, which would be expected to affect the three resonances differently.

We note that no degradation was observed in the activity of the SABRE catalyst over the one hundred repeat experiments shown in Figure 4. We routinely repolarize a single sample over a full day of experiments without any evident sample aging. While the activated SABRE catalyst is known to be air-sensitive, our setup ensures that an inert atmosphere is maintained throughout. However, the stability of the SABRE catalyst can be affected by the choice of solvent or target analyte(s), and hydrogen isotope exchange has been observed for SABRE samples in deuterated solvents, limiting the effective sample lifetime.^33^

**Figure 4:**
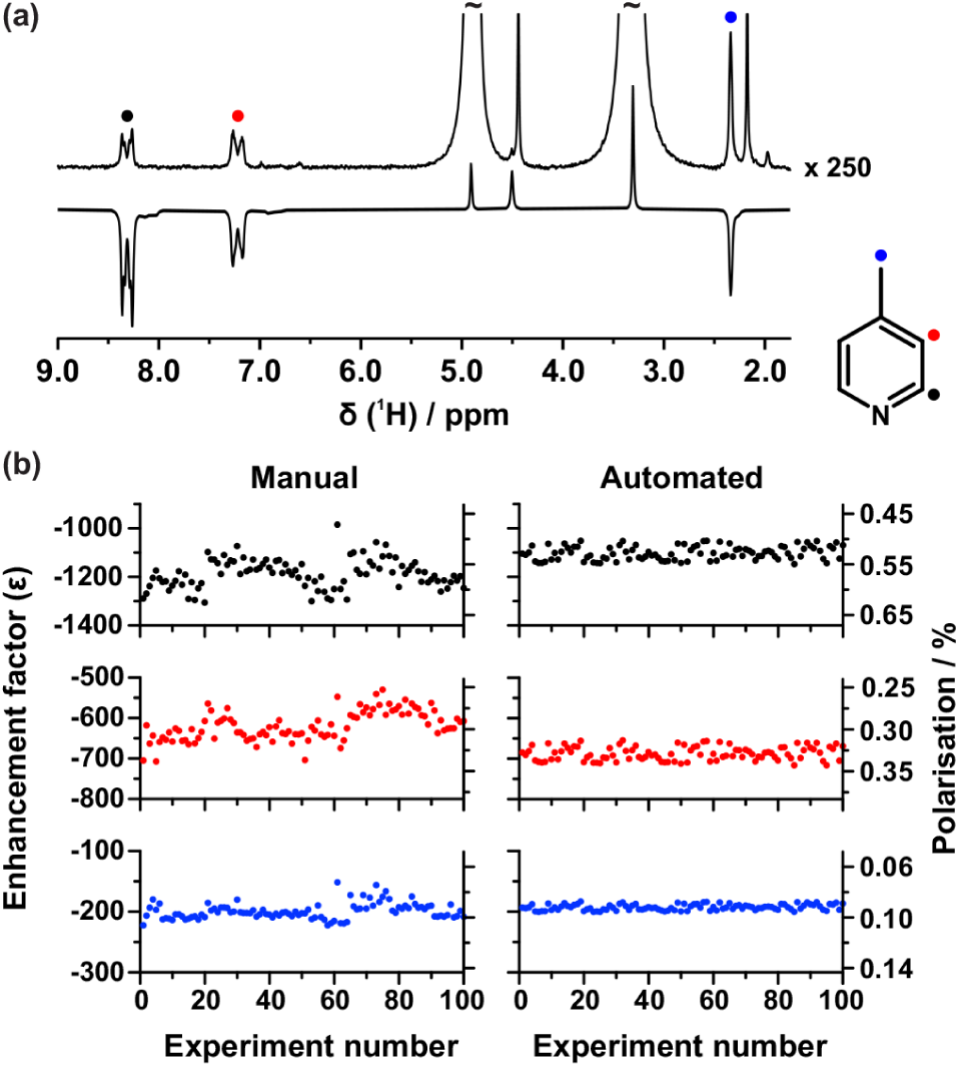
(**a**) Single-scan, thermally polarized (top, × 250) and hyperpolarized (bottom) ^1^H NMR spectra of 4-methylpyridine (100 mM) with [IrCl­(COD)­(IMes)] SABRE precatalyst (5 mM) in methanol, collected at 1.4 T (60 MHz). Hyperpolarization was generated using the automated SABRE workflow. (**b**) Integral enhancement factors (ϵ) and polarization levels for the *ortho* (top), *meta* (middle), and methyl (bottom) signals across 100 successive experiments, where SABRE hyperpolarization was generated using the manual shake-and-drop approach (left) and using the automated SABRE workflow (right). Enhancement factors are calculated as the ratio of the peak integral with and without hyperpolarization. Polarization percentages are calculated by multiplying the enhancement factors by the polarization at thermal equilibrium at *B*_0_ = 1.4 T (4.8 ppm).

### Automated SABRE for the Analysis of Mixtures

To demonstrate the utility of the automated SABRE method for mixture analysis, 3 mM each of the well-established SABRE substrates pyridine, 4-methylpyridine, and 3,5-dimethylpyridine^43,80^ with 600 μM of the SABRE precatalyst in methanol, was studied. While the single-scan SABRE-hyperpolarized ^1^H NMR spectrum (Figure 5a) of this mixture exhibits narrow line widths and strong signal enhancements across all three components, extensive signal overlap, particularly in the aromatic region (δH1=6.5−8.0ppm) , makes spectral analysis challenging.

**Figure 5:**
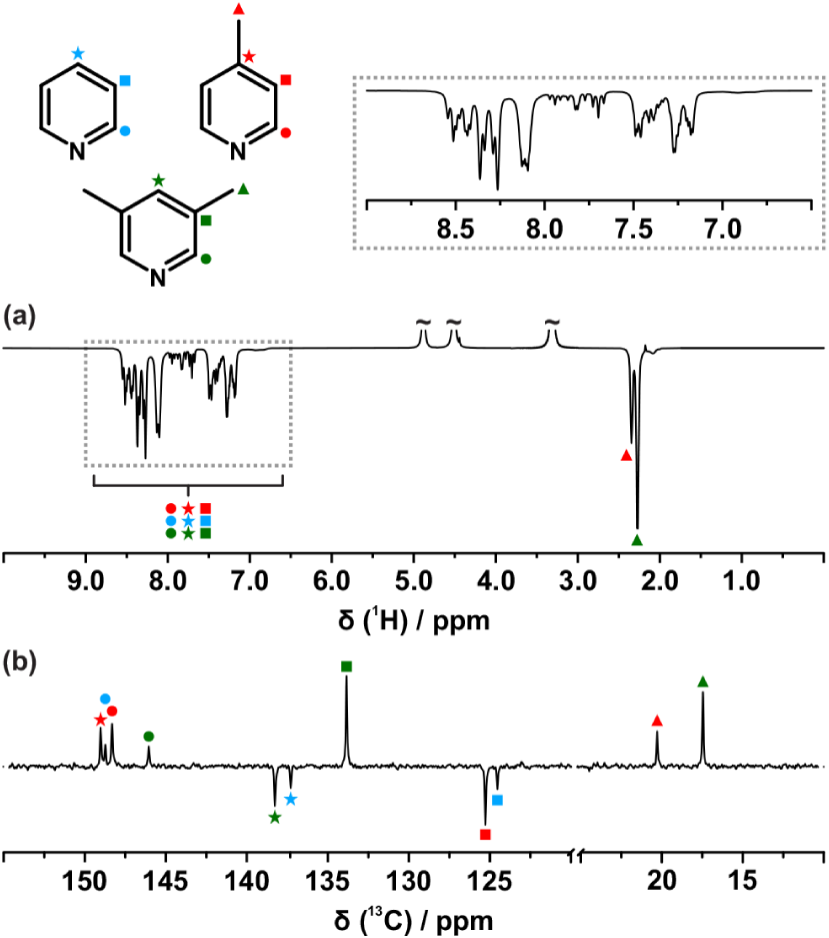
(**a**) Single-scan SABRE-hyperpolarized ^1^H NMR spectrum of pyridine, 4-methylpyridine, and 3,5-dimethylpyridine (3 mM each) with 600 μM [IrCl­(COD)­(IMes)] SABRE precatalyst in methanol. Inset: Expansion of the aromatic region: (δH1=6.5−8.0ppm) . (**b**) Corresponding SABRE-enhanced ^13^C­{^1^H} NMR spectrum of the same sample, collected using 64 scans. ^13^C signal enhancement was generated using INEPT optimized for three-bond coupling following ^1^H polarization at *B*_PTF_ = 6.4 mT.

One solution to this spectral congestion is to exploit the wider chemical shift range of the spin-dilute ^13^C nucleus. For the three-component mixture, optimal ^13^C signal enhancement was obtained using INEPT with a transfer delay (1/4*J*_CH_) optimized for a three-bond coupling (^3^ *J*_CH_ = 10.5 Hz). Even though INEPT provides efficient ^13^C hyperpolarization, some of the ^13^C­{^1^H} NMR signals obtained from low-concentration analytes at natural abundance (3 mM in this case) remain too weak to be distinguished from noise in a single scan. In the 64-scan (18 min) ^13^C­{^1^H} NMR spectrum of the mixture (Figure 5b), all signals are clearly discernible, with SNR values ranging from 8 to 40. The SABRE-enhanced INEPT experiment used here relies on first hyperpolarizing ^1^H nuclei directly bonded to ^12^C at *B*_PTF_ = 6.4 mT, and then transferring this hyperpolarization to ^13^C nuclei on the same molecule through weaker multibond couplings. A key benefit of this approach is that it allows the observation of quaternary carbon signals, which are typically absent in ^13^C­{^1^H} INEPT spectra when a one-bond transfer is used. Our previously published single-scan ^13^C­{^1^H} SABRE-INEPT spectrum of 260 mM 4-methylpyridine (1 T detection field, manual sample transfer) yielded SNR values between 23 and 91.^52^ After correcting for differences in concentration, number of scans, and *p*-H_2_ enrichment, the current results demonstrate at least a 10-fold improvement in hyperpolarization efficiency (the full comparison is provided in the Supporting Information).

Hyperpolarized ^13^C­{^1^H} benchtop NMR spectra have also been demonstrated by Kircher et al. using the *in situ* SLIC-SABRE method.^62^ Two single-scan spectra of 60 mM 4-aminopyridine produced SNR values of 143 and 66 for the *ortho* and *meta* ^13^C resonances, respectively. No hyperpolarization was observed for the *para* carbon due to the negligible five-bond scalar coupling to the *p*-H_2_-derived hydride ligands in the SABRE catalyst. A comparison of the average SNR values, again accounting for the differences in sample concentration, number of scans, and *p*-H_2_ enrichment level, suggests that the observed hyperpolarization using our approach is roughly comparable to that obtained using the *in situ* method (see the Supporting Information for the full comparison), with the *in situ* method performing better for the *ortho* carbon and our *ex situ* method performing better for all other resonances. This highlights an important benefit of the *ex situ* approach. Due to the indirect nature of the transfer, all resonances of each component of the mixture receive significant polarization transfer within a single experiment and without requiring resonance-specific optimization of the polarization transfer conditions. However, transferring polarization *in situ* allows for shorter repolarization times (6 s) and hence more efficient signal averaging and does not require a sample-shuttling apparatus.

The greater ^13^C signal dispersion can be exploited to disentangle the overlapping signals in the aromatic region of the ^1^H NMR spectrum using a two-dimensional (2D) ^1^H–^13^C HETCOR experiment. Figure 6a presents a 2D ^1^H–^13^C HETCOR spectrum of the same mixture of substrates as in Figure 5, acquired using 8 scans and 32 increments in 2 h 25 min. Multibond correlations are observed for the various resonances of pyridine, 4-methylpyridine, and 3,5-dimethylpyridine, enabling the assignment of the corresponding ^1^H NMR signals. Notably, the high resolution in the ^13^C dimension enables the assignment of ^1^H peaks even in the highly overlapped aromatic region, and despite the relatively low resolution in the indirect ^1^H dimension.

**Figure 6:**
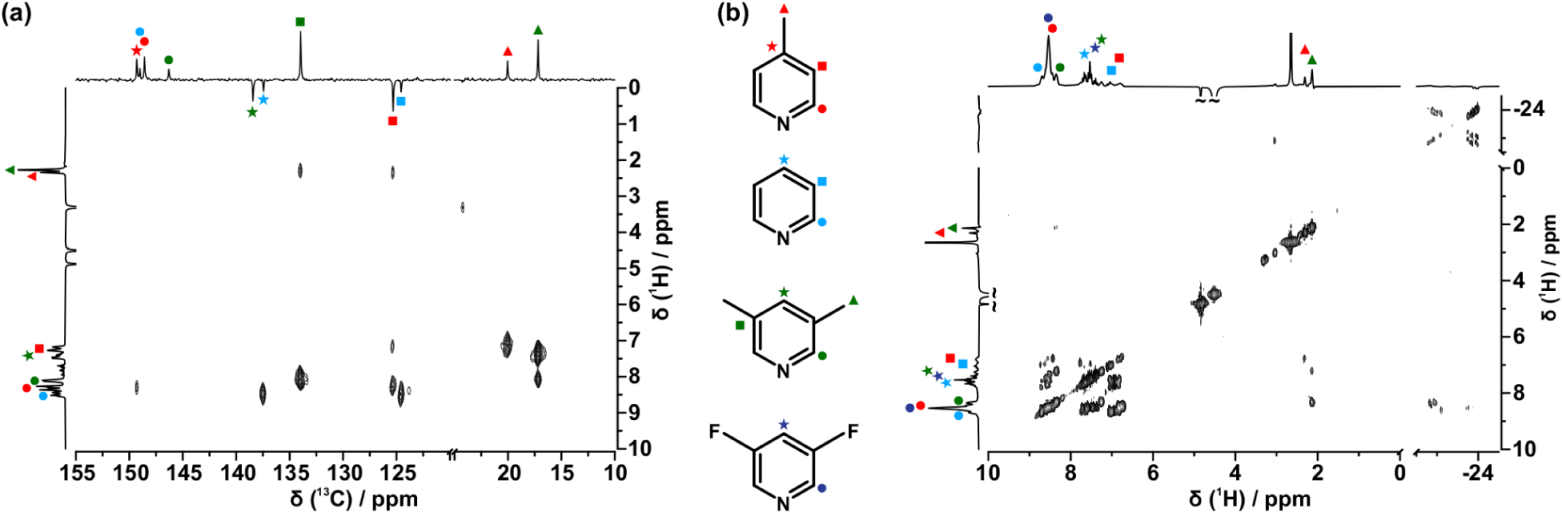
(**a**) 2D SABRE-HETCOR spectrum of pyridine, 4-methylpyridine, and 3,5-dimethylpyridine (3 mM each) with 600 μM SABRE precatalyst in methanol. (**b**) 2D SABRE-COSY spectrum of pyridine, 4-methylpyridine, 3,5-dimethylpyridine, and 3,5-difluoropyridine (750 μM each) with 25 mM DMSO and 5 mM SABRE precatalyst in methanol-*d*_4_.

Although the heteronuclear detection strategies employed in Figures 5 and 6a are effective at resolving signal overlap, the low natural abundance (1.1%) of ^13^C inherently restricts its practical detection limit, even with hyperpolarization. For the analysis of mixtures at lower (submillimolar) concentrations, the higher sensitivity of ^1^H detection is beneficial.

In moving to lower concentration mixtures at a fixed precatalyst concentration, there no longer exists a large combined excess of substrate to drive the formation of the active SABRE catalyst (Figure 1), a species which requires the simultaneous coordination of three substrate molecules. Instead, the weakly binding solvent (present in overwhelming majority) competes effectively for the iridium binding sites, which shifts the equilibrium away from the SABRE-active tris-substrate species toward solvent-coordinated complexes, reducing hyperpolarization efficiency.^81^ To address this, a cosubstrate can be introduced into the mixture to occupy the vacant coordination sites on the catalyst. This stabilizes the catalyst and promotes efficient polarization transfer to species present at substoichiometric levels, extending the ^1^H benchtop NMR detection limit toward the nanomolar regime.^49,65^

The utility of 2D homonuclear correlation NMR experiments in mixture analysis is highlighted by the SABRE-COSY spectrum (Figure 6b) of a sample containing 750 μM each of pyridine, 4-methylpyridine, 3,5-dimethylpyridine, and 3,5-difluoropyridine. In this mixture, the SABRE catalyst (5 mM) is present in excess relative to the combined concentration of substrates, and so dimethyl sulfoxide (DMSO, 25 mM) is used as a stabilizing cosubstrate. The resonances of 4-methylpyridine and 3,5-dimethylpyridine are easily assigned based on correlations between their methyl and aromatic resonances. The remaining signals can then be distinguished as either pyridine or 3,5-difluoropyridine by noting the absence of correlations to the aromatic *meta* signal for the fluorinated species.

Unlike conventional COSY spectra acquired at thermal equilibrium, SABRE-enhanced COSY spectra often appear to be asymmetric about the diagonal.^82^ This can provide insight into the underlying polarization transfer dynamics. In Figure 6b, cross-peaks corresponding to magnetization transfer from aromatic to methyl protons of 4-methylpyridine and 3,5-dimethylpyridine are significantly more intense than those for the reverse transfer. This disparity arises because aromatic protons are hyperpolarized more efficiently by the SABRE process than aliphatic protons, owing to their closer proximity to the *p*-H_2_-derived hydride ligands when bound to the SABRE catalyst.

The reduced frequency dispersion inherent to the lower field strength of benchtop NMR detection enables the practical sampling of a wide spectral width in the indirect dimension of the COSY experiment. This facilitates the observation of the *p*-H_2_-derived hydride ligands of SABRE-active complexes in solution, centered around −23.5 ppm. Interestingly, correlations are observed between the *ortho* protons of each component of the mixture with a single peak in the hydride region. This indicates that the SABRE signals observed for this mixture correspond predominantly to hyperpolarized molecules bound to the SABRE catalyst and not in free solution. Indeed, SABRE-enhanced NMR spectra of each individual component exhibit differences in chemical shift when compared to the molecule in free solution (Figures S19 and S20). The observation of a single hyperpolarized species for each component of the mixture is beneficial as it reduces peak overlap. However, it may pose a challenge for complex mixture analysis because peaks cannot necessarily be assigned using databases of known chemical shifts for each analyte in free solution.

## Conclusions

We have presented a robust system for automated SABRE hyperpolarization that integrates into the recycle delay of any NMR pulse sequence, achieving typical repolarization times of 13 s. The signal enhancement factors and repeatability (2.5% over 100 experiments) improve upon previous flow-based systems^30,52^ and match other mechanical transfer methods.^69,70^ This stability enabled signal averaging for natural abundance ^13^C­{^1^H} benchtop NMR spectroscopy of a low-concentration (3 mM each component) mixture, where long-range INEPT transfer effectively enhanced all ^13^C resonances and yielded SNRs ranging from 8 to 40 in 18 min. A key advantage of this field-cycled SABRE approach, compared with *in situ* methods such as SLIC-SABRE,^62^ is the ability to observe all ^13^C resonances in a single experiment.

The indirect hyperpolarization of ^13^C also enabled the acquisition of a 2D ^1^H–^13^C HETCOR spectrum, which facilitated the assignment of the highly overlapped aromatic region in the ^1^H NMR spectrum of the 3 mM mixture. We also demonstrated that hyperpolarized homonuclear 2D experiments, such as COSY, can resolve peak overlap in mixtures of species present at submillimolar concentrations. Our approach has potential applications in metabolic analysis, building on recent developments in the analysis of cell cultures^83^ and biofluids^70^ using SABRE-enhanced benchtop NMR. For example, while Fleischer et al. recently detected and quantified the metabolite nicotinamide using SABRE-enhanced benchtop NMR, they could not isolate other components of the complex urine extract mixture due to spectral congestion.^70^ 2D experiments could be used to expand this approach, enabling the identification of other urinary metabolites. Alternatively, combining automated SABRE with advanced high-resolution techniques, such as pure shift^84,85^ or ultraselective observation,^86^ which typically suffer from sensitivity losses, could simultaneously address the challenges of low sensitivity and signal overlap, enabling new analytical applications of benchtop NMR spectroscopy.

The range of molecules that can be enhanced using the automation setup could be expanded through the use of SABRE-Relay.^34,87^ In this approach, a polarization carrier with labile protons, typically an amine, is used to enhance target analytes in solution via proton exchange, thus hyperpolarizing molecules that do not bind reversibly to the catalyst. The main challenge in implementing SABRE-Relay is the selection of a suitable solvent. Methanol, used herein, contains exchangeable protons and therefore acts as a polarization sink in SABRE-Relay experiments, while dichloromethane, the standard SABRE-Relay solvent, has a low boiling point and is thus not practical to use with the bubbling apparatus.

## Supplementary Material



## Data Availability

The data supporting this research, including all raw NMR data and acquisition parameters, example pulse programs, microcontroller source codes, hardware control macros, and stereolithography files for 3D-printed components, are openly available from the research data repository of the University of York at 10.15124/d941c05a-ab67-4da1-8382-6bd0eb3ae39d.

## Notes

Full experimental details; bills of materials; technical drawings; and additional NMR spectra (PDF)

## §

Gold Standard Phantoms Ltd., Sheffield, South Yorkshire, S9 4WQ, United Kingdom
