# Supplementary material for: Automated para-Hydrogen Hyperpolarization for Mixture Analysis Using 1H and 13C Benchtop NMR Detection
Source: Anal Chem. 2026 Jun 20;98(25):18713–21. doi: 10.1021/acs.analchem.6c01237 (PMC13325455; doi:10.1021/acs.analchem.6c01237)
Supplement: Supplementary file 1 [file ac6c01237_si_001.pdf]

Supporting Information

Automated *para*hydrogen hyperpolarisation for mixture analysis using ^{1}H and ^{13}C benchtop NMR detection

Daniel A. Taylor,*[a] James McCall,[a] Fraser Hill-Casey,[a,b] Jonathan Hedges,[c] Izzy Hehir,[a] Gregory J. Yule,[a] Stuart Murray,[a] Abigail Mortimer,[a] and Meghan E. Halse*[a]

[a]Department of Chemistry, University of York, Heslington, York, North Yorkshire, YO10 5DD, United Kingdom

[b]Current address: Gold Standard Phantoms Ltd, Sheffield, South Yorkshire, S9 4WQ, United Kingdom

[c]Research IT, University of York, Heslington, York, North Yorkshire, YO10 5DD, United Kingdom

*Email: daniel.a.taylor@york.ac.uk, meghan.halse@york.ac.uk

Contents

S1. Experimental details S2

S2. *Para*hydrogen generation apparatus S4

S3. Solenoid valve manifold S8

S4. Custom NMR tube and gas adaptor S18

S5. Linear actuator S22

S6. Polarisation transfer field S26

S7. SABRE-enhanced $^{13}C\{^{1}H\}$ benchtop NMR SNR comparison S28

S8. Single-component spectra S30

S9. Repository contents S34

S10. References S35

S1. Experimental details

For manual "shake-and-drop" SABRE experiments, 800 µL of the sample was transferred to an NMR tube equipped with a J Young valve. The sample was degassed over three freeze-pump-thaw cycles using liquid nitrogen. For each experiment, the headspace of the NMR tube was filled with 4 bar (absolute) of 51 % *para*hydrogen (pH_2), generated using a home-built pH_2 converter (**Section S2**). The sample was subsequently shaken for 10 s at the polarisation transfer field (B_{PTF}), before it was transferred to the NMR spectrometer for data collection. Typical sample transfer times were on the order of 2 s. B_{PTF} was generated using a handheld permanent magnet Halbach array with an average field of 6.2 mT.[1] Between each measurement, the pH_2 in the sample was refreshed by evacuating the headspace of the NMR tube and refilling with fresh pH_2. Before collecting data, the fill-shake-acquire process was repeated ten times to activate the SABRE catalyst *via* H_2 addition.

For automated SABRE experiments, 800 µL of the sample was transferred to a bespoke NMR tube fitted with a custom gas delivery adaptor (**Section S4**). The adaptor was connected to both the valve manifold (**Section S3**) and the linear actuator (**Section S5**). Upon execution of each experiment, the sample was automatically positioned at B_{PTF}, before pH_2 (51 %) was bubbled through the solution at a pressure of 5.5 bar (absolute) and a flow rate of 100 mL min^{-1}. After 10 s, bubbling was stopped and the sample was automatically shuttled to the NMR spectrometer for data collection. The sample transfer time was 1.27 s in all experiments, and an additional 230 ms delay was included for sample stabilisation before executing the NMR pulse sequence. B_{PTF} was generated using a permanent magnet Halbach array mounted on top of the NMR spectrometer, with an average field of 6.4 mT (**Section S6**). Before collecting data, the bubble-shuttle-acquire process was repeated ten times to activate the SABRE catalyst *via* H_2 addition.

NMR measurements were performed using a 62 MHz (1.4 T) Magritek Spinsolve 60 Ultra spectrometer equipped with a dual-channel Multi-X probe. Shimming and frequency calibration was performed on a reference sample containing 5 % H_2O in D_2O before data was collected. For repeatability studies (**Fig. 4**), the first and second order shims were optimised (using a 1 min 15 sec procedure) to maintain linewidth by shimming on the sample after every 10 experiments. Data was acquired using

SpinsolveExpert v2.02.14 and processed using MestReNova v16.0.0. Raw experimental data, acquisition parameters, pulse programs and macros for controlling the NMR experiment, valve manifold and linear actuator are openly available from the research data repository of the University of York (DOI: 10.15124/d941c05a-ab67-4da1-8382-6bd0eb3ae39d).

S2. *Para*hydrogen generation apparatus

The home-built *para*hydrogen converter consists of an ErreDue Mars 600N hydrogen generator (10.5 bar maximum operating pressure, 640 mL min^{-1} maximum flow rate) connected to a series of Swagelok® valves and components. *Para*hydrogen (~51 %) is generated on-demand inside a copper coil that is packed with hydrated iron(III) oxide (Sigma-Aldrich) when cooled with liquid nitrogen to 77 K. The coil is flanked by 0.5 µm in-line particulate filters to prevent iron(III) oxide leaving the coil and warming to room temperature, where it would catalyse the back-conversion of *para*hydrogen to *ortho*hydrogen. Gas can be directed either to an adaptor for pressurisation of the headspace of an NMR tube fitted with a J Young valve, or to the solenoid valve manifold (**Section S3**) for bubbling of gas through an NMR sample. The system is equipped with a proportional relief valve for safety and a digital pressure gauge (SMC) for pressure monitoring. **Figure S1** provides a schematic of the system. **Figures S2** and **S3** provide photographs of the system. **Table S1** provides a parts list for replication of the system.

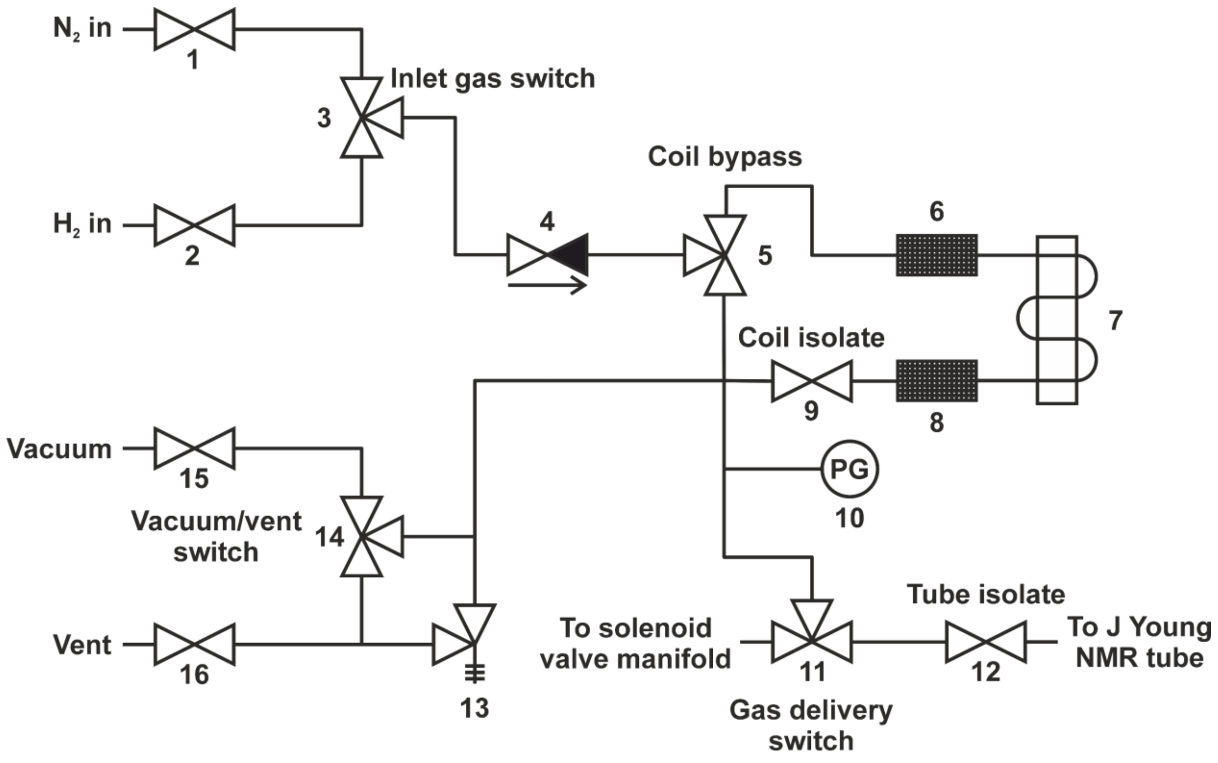

Figure S1: Schematic of the home-built *para*hydrogen converter.

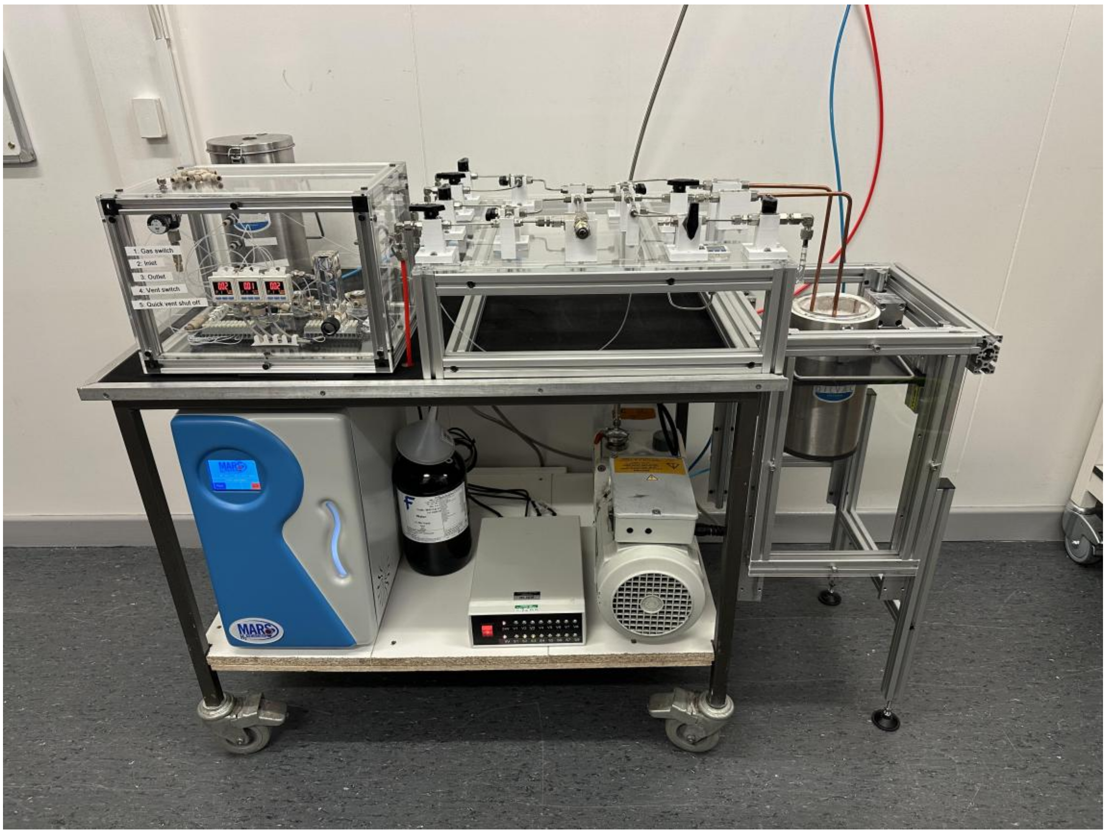

Figure S2: Front view photograph of the home-built *para*hydrogen converter.

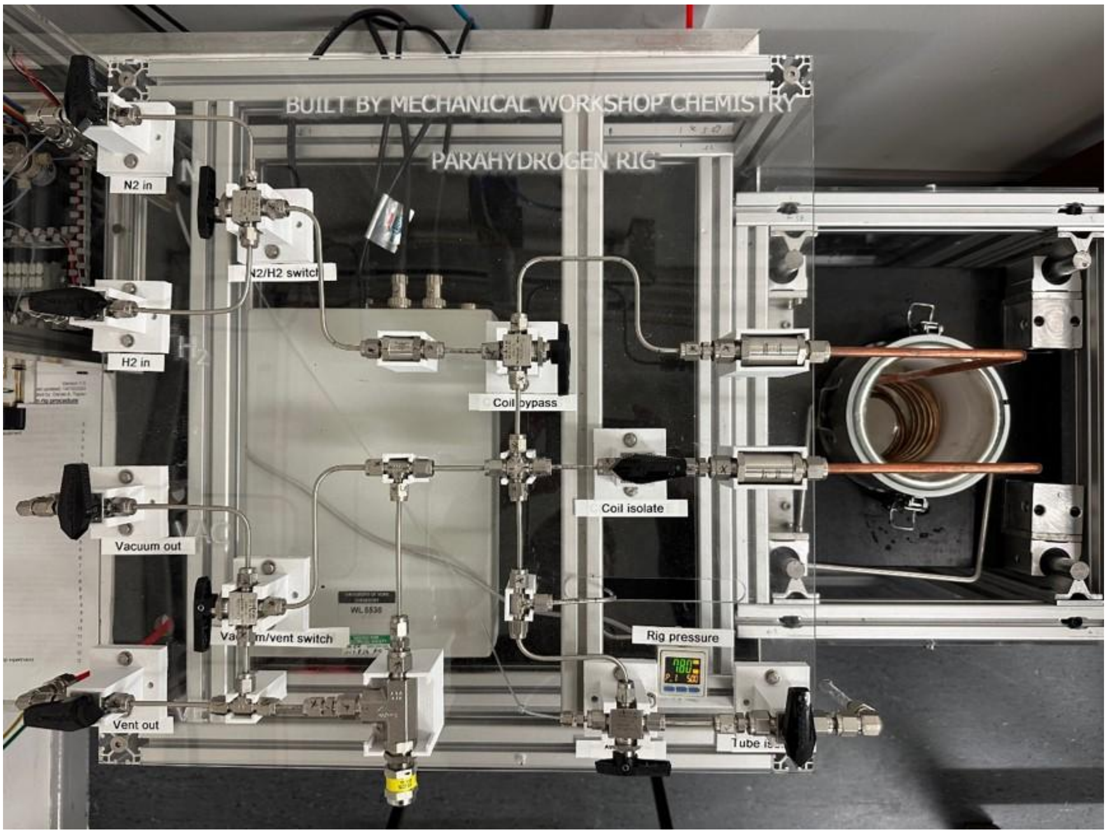

Figure S3: Top view photograph of the home-built *para*hydrogen converter.

Table S1: Bill of materials for the home-built *para*hydrogen converter.

Manufacturer	Product code	Quantity	Unit price / £ (excl. VAT)	Vendor	Function/Description
Chemglass	CG-306-03	1	88.49	GPE Scientific	J Young NMR tube adaptor
Dilvac	DEW1012	1	183.94	Scientific Laboratory Supplies	**7**: Liquid nitrogen dewar
Edwards	A65201903	1	2028.00	WolfLabs	Vacuum pump
ErreDue	MARS 600 N	1	5995.00	GPE Scientific	H_2 generator
Sigma-Aldrich	371254	50 g	118.00	Merck	**7**: Spin isomer interconversion catalyst
SMC	ISE20B-R-01-W	1	122.56	RS Components	**10**: Digital pressure gauge
Swagelok	SS-2P4T-BK	6	86.14	Swagelok	**1**: Controls the flow of N_2 into the system<br>**2**: Controls the flow of H_2 into the system<br>**9**: Isolates coil (**7**) from the system<br>**12**: Isolates NMR tube from the system<br>**15**: Controls evacuation of the system<br>**16**: Controls venting of the system
Swagelok	SS-41GXS2	4	120.06	Swagelok	**3**: Switches H_2/N_2 inlet gases<br>**5**: Bypasses coil (**7**)<br>**11**: Switches gas delivery mode<br>**14**: Switches between evacuation/venting
Swagelok	SS-4F-05	2	84.47	Swagelok	**6**/**8**: Prevents iron(III) oxide leaving coil (**7**)
Swagelok	SS-2C-1	1	74.12	Swagelok	**4**: Prevents gas backflow to inlet
Swagelok	SS-RL3S4	1	236.56	Swagelok	**13**: Pressure relief valve
Swagelok	SS-200-3	3	31.74	Swagelok	Union tee for branching to pressure gauge (**10**) and pressure relief valve (**13**)
Swagelok	SS-200-4	1	56.98	Swagelok	Union cross for connecting components of system

Swagelok	SS-4BHT-72	4	106.95	Swagelok	Tubing for connecting H_2/N_2 inlets and vacuum/vent outlets to system
Swagelok	SS-400-R-2	6	15.87	Swagelok	Reducers for connecting H_2 inlet tubing to H_2 generator and system, N_2 inlet tubing, vacuum/vent outlet tubing, and J Young NMR tube adaptor to system
Swagelok	SS-200-R-4	4	11.85	Swagelok	Reducers for connecting in-line particulate filters (**6**/**8**) and pressure relief valve (**13**) to system
Swagelok	SS-100-R-2	1	17.37	Swagelok	Reducer for connecting solenoid valve manifold (**Section S2**) to system
Swagelok	SS-200-9	5	19.55	Swagelok	Union elbow for connecting H_2/N_2 inlet tubing, vacuum/vent outlet tubing and J Young NMR tube adaptor to system
Swagelok	SS-T2-S-035-6ME	6 m	69.54	Swagelok	1/8” 316 stainless steel tubing for connection of components of system
Thorite	THH14	3 m	20.83	Thorite	**7**: ¼” O.D. half-hard copper tubing

S3. Solenoid valve manifold

The solenoid valve manifold consists of a series of NResearch three-way isolation valves connected by 1/16“ O.D. perfluoroalkoxy tubing. Gas from the *para*hydrogen converter (**Section S2**) is pneumatically regulated at the manifold inlet before it is delivered to the NMR sample. Gas flow through the sample is controlled using a variable area flow meter. Digital pressure gauges (SMC) monitor gas pressure at the manifold inlet, across the NMR tube, and at the manifold outlet. The valves in the manifold are connected such that gas can be routed through both the inlet and outlet ports of the NMR tube for initial pressurisation. This reduces bulk sample motion upon pressurisation and minimises the risk of chemical contamination of the manifold. A 2 mL solvent reservoir is incorporated before the NMR tube to pre-saturate inlet gas with solvent. This compensates for solvent evaporation from the sample during gas bubbling and extends sample lifetimes. **Figure S4** provides a schematic of the system. **Figures S5**, **S6**, and **S7** provide photographs of the wetted components of the system. **Table S2** provides a parts list for the wetted components of the system.

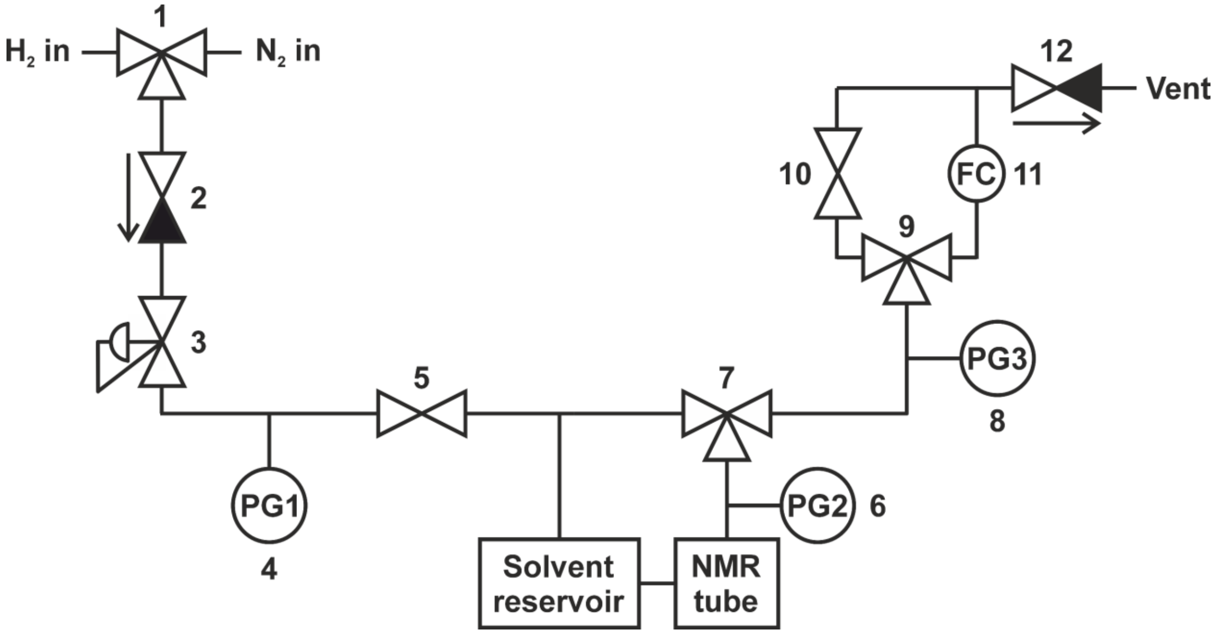

Figure S4: Schematic of the solenoid valve manifold.

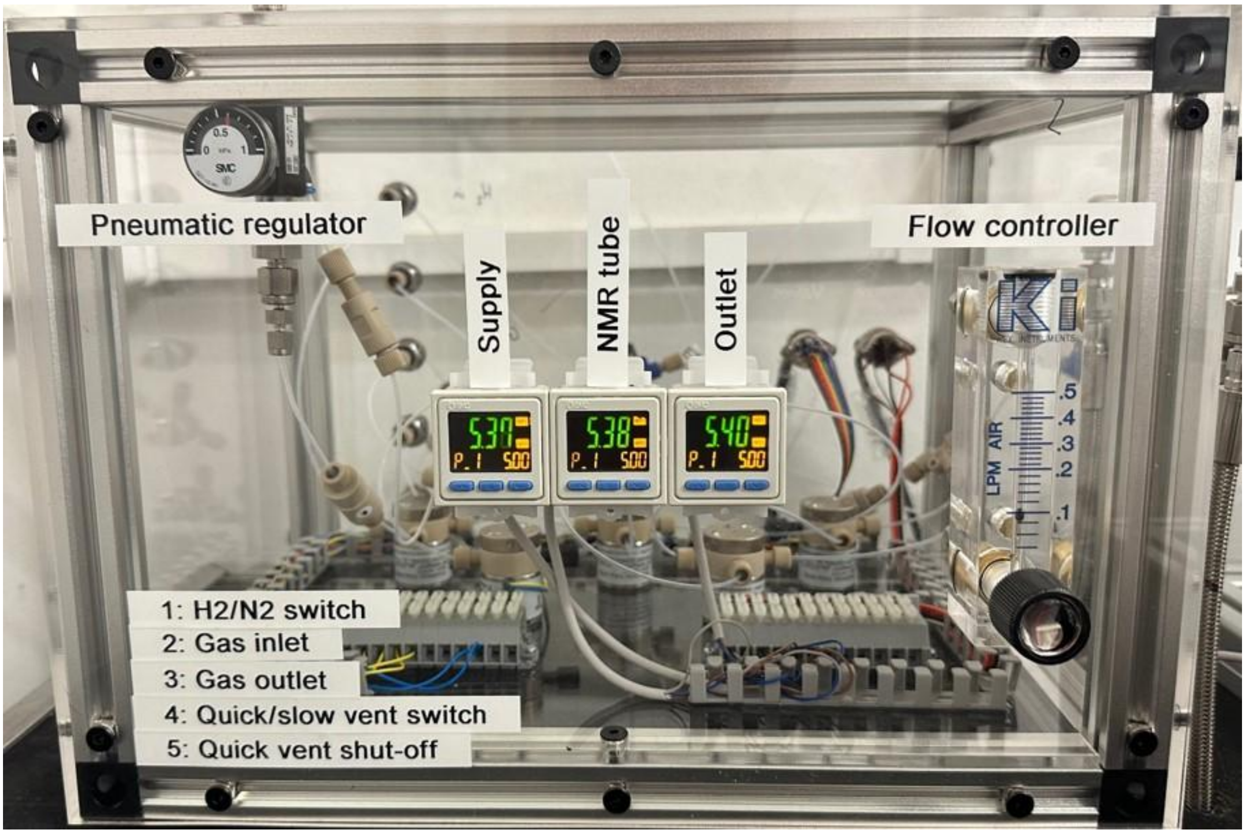

Figure S5: Front view photograph of the solenoid valve manifold.

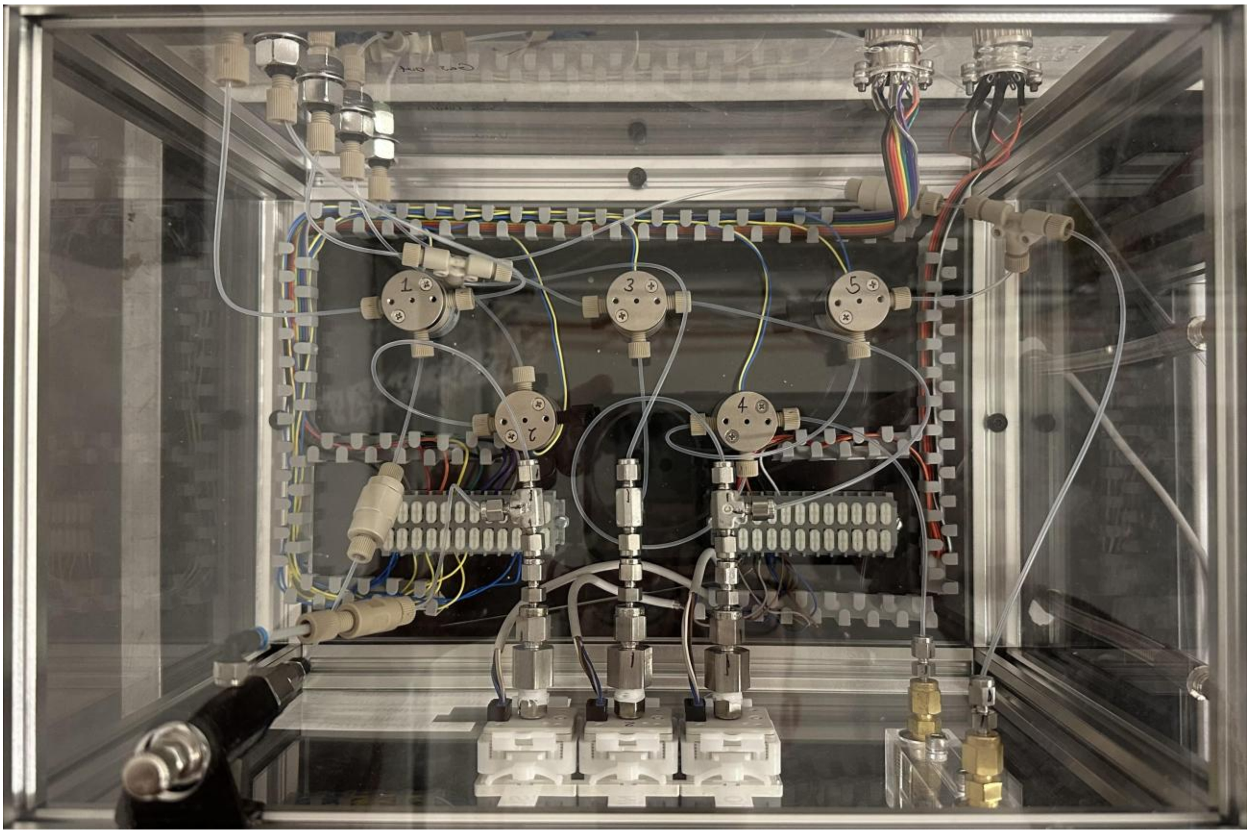

Figure S6: Top view photograph of the solenoid valve manifold.

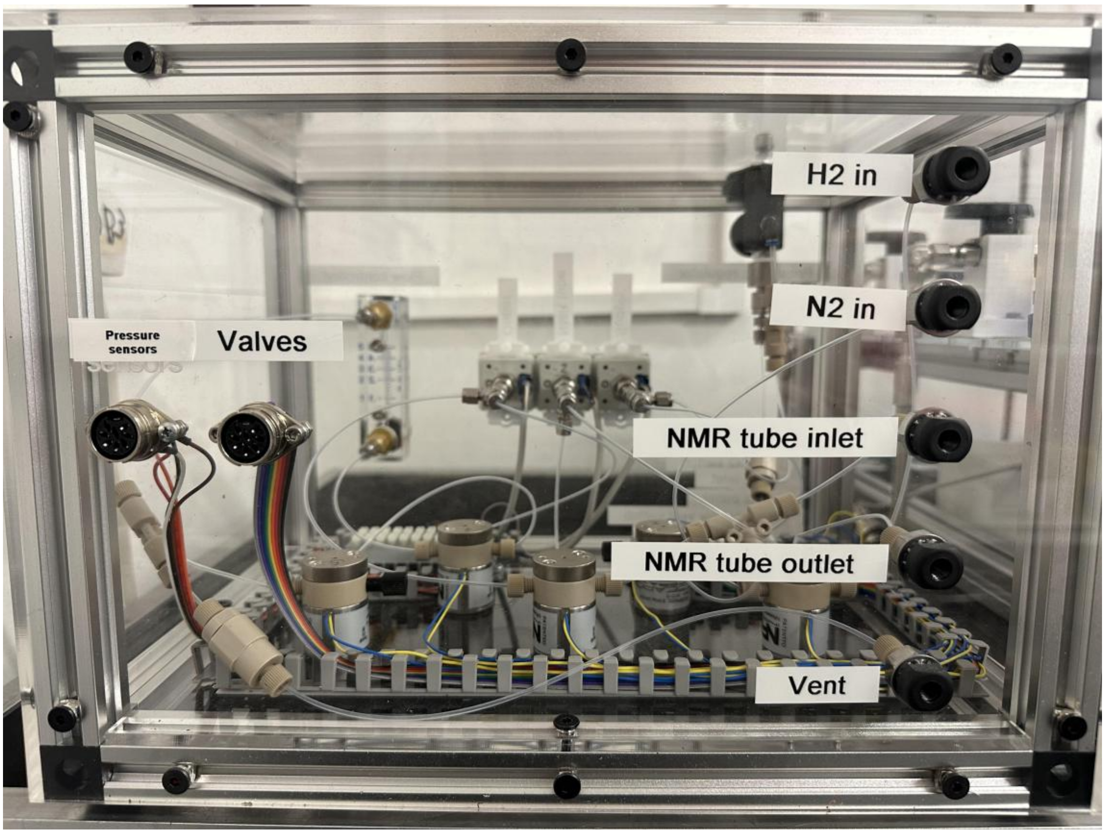

Figure S7: Rear view photograph of the solenoid valve manifold.

Table S2: Bill of materials for the wetted components of the solenoid valve manifold.

Manufacturer	Product code	Quantity	Unit price / £ (excl. VAT)	Vendor	Function/Description
3M	3811/16	2	4.91	Onecall	Ribbon cable for connecting Wago DIN rail terminal blocks to DIN sockets
Festo	QSML-M5-3	1	32.00 (10/PK)	RS Components	Fitting for connecting 1/8" O.D. tubing to outlet of pneumatic regulator (**3**)
IDEX	1507L	1	170.35	Cole-Parmer	PFA tubing, 1/16" O.D. x 0.040" I.D.
IDEX	1509L	1	288.93	Cole-Parmer	PFA tubing, 1/8" O.D. x 0.062" I.D.
IDEX	CV-3330	2	138.26	Cole-Parmer	**2**/**12**: Check valve
IDEX	P-255	29	40.20 (10/PK)	Cole-Parmer	Super Flangeless™ nuts for connecting tubing to manifold components
IDEX	P-259	29	38.94 (10/PK)	Cole-Parmer	Super Flangeless™ ferrules for connecting 1/16" O.D. tubing to manifold components
IDEX	P-309	2	1.66	Cole-Parmer	Port plugs for valves (**5**/**10**), where three-way isolation is unnecessary
IDEX	P-331	1	4.74	Cole-Parmer	Super Flangeless™ nut for connecting 1/8" O.D. tubing to manifold components
IDEX	P-359	1	4.36	Cole-Parmer	Super Flangeless™ ferrule for connecting 1/8" O.D. tubing to manifold components
IDEX	P-441BLK	5	19.24	Cole-Parmer	Bulkhead unions for connecting the solenoid valve manifold to the *para*hydrogen converter (**Section S1**), house nitrogen supply, NMR tube inlet, NMR tube outlet, and vent
IDEX	P-702-01	1	15.15	Cole-Parmer	Union for connecting 1/8" O.D. tubing from the outlet of pneumatic regulator (**3**) to the 1/16" O.D. tubing used in the rest of the manifold.
IDEX	P-712-01	2	25.31	Cole-Parmer	Union tees

Instruments Direct	2510A2A12BVBN	1	96.57	RS Components	**11**: Variable area flow meter
NResearch	HP225PK032	5	113.90	NResearch	**1**/**5**/**7**/**9**/**10**: Three-way isolation valves
PrehKeyTec	71206-070/0800	1	4.43	Onecall	7-pin DIN socket for connecting digital pressure gauges to electrical components
PrehKeyTec	71206-080/0800	1	4.15	Onecall	8-pin DIN socket for connecting isolation valves to electrical components
SMC	ARJ210-M5BG	1	34.05	RS Components	**3**: Pneumatic regulator
SMC	ISE20B-R-01-W	3	126.24	RS Components	**4**/**6**/**8**: Digital pressure gauges
SMC	ZS-46-B	3	2.24	RS Components	Panel mount adaptors for digital pressure gauges
Swagelok	B-100-1-2	2	7.48	Swagelok	Fitting for connecting 1/16" O.D. tubing to the inlet and outlet of variable area flow meter (**11**)
Swagelok	SS-100-3	3	51.41	Swagelok	Union tees for connecting 1/16" O.D. tubing to digital pressure gauges (**4**/**6**/**8**)
Swagelok	SS-100-R-2	4	17.37	Swagelok	Reducer for connecting 1/16" O.D. tubing to inlet of pneumatic regulator (**3**), and to digital pressure gauges (**4**/**6**/8)
Swagelok	SS-101-PC	3	15.47	Swagelok	Port connectors for connecting 1/16" O.D. tubing to digital pressure gauges (**4**/**6**/**8**)
Swagelok	SS-200-7-2RT	4	12.88	Swagelok	Fitting for connecting 1/16" O.D. tubing to inlet of pneumatic regulator (**3**), and to pressure gauges (**4**/**6**/**8**)
Wago	261-112/341-000	2	5.43	Onecall	DIN rail terminal blocks for connecting valves and digital pressure sensors to electrical components

The solenoid valves are controlled by an Arduino® compatible Whadda ATmega2560 microcontroller that is connected by USB to the NMR spectrometer control PC. In standard operation, the PC sends single characters over this serial connection to trigger valve switching. Alternatively, where available, transistor-transistor logic (TTL) pulses can be sent directly from the NMR spectrometer to the microcontroller to trigger valve switching. The microcontroller also interfaces with the three digital pressure sensors in the solenoid valve manifold, as well as the digital pressure sensor in the *para*hydrogen converter, to enable pressure logging and monitoring on the PC.

The microcontroller is housed in a separate enclosure isolated from the wetted components of the solenoid valve manifold. This enclosure additionally contains a 24 V DC power supply (for power to valves/pressure sensors), a 24/9 V DC/DC converter (for power to microcontroller), five valve driver cards, and 16 LEDs (mounted to the front panel) for valve state indication. The rear panel features 8-pin and 7-pin DIN sockets to provide power and communication to the valves and digital pressure sensors in the manifold, a 4-pin M12 socket to provide power and communication to the digital pressure sensor in the *para*hydrogen converter, a 5-pin DIN socket for TTL inputs, a USB B socket for connection of the microcontroller to the spectrometer control PC, and a C14 socket for 230 V input voltage. **Figure S8** provides a pinout diagram for the microcontroller. **Figures S9**, **S10**, and **S11** provide photographs of the electronic components of the system. **Table S3** provides a parts list for the electronic components of the system.

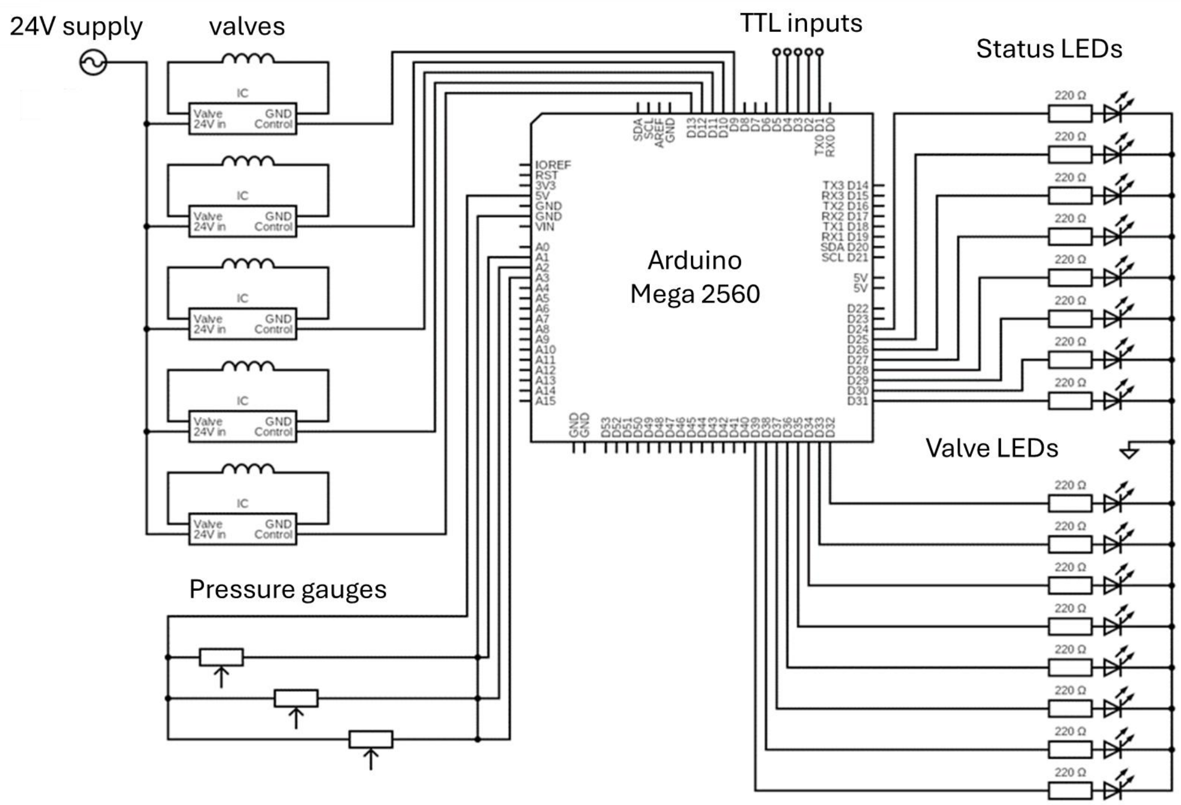

Figure S8: Pinout diagram for the Whadda ATmega2560 microcontroller.

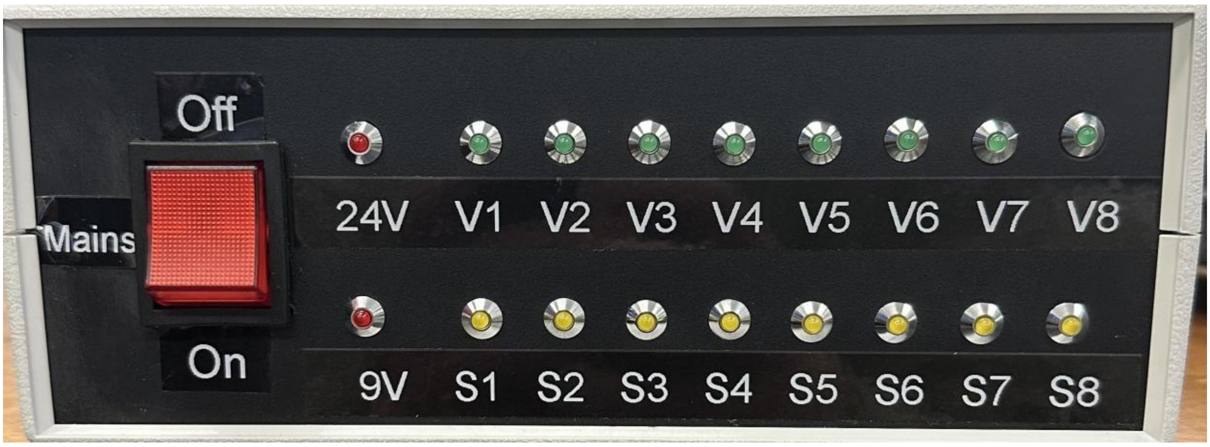

Figure S9: Front view photograph of the electronics enclosure for the solenoid valve manifold.

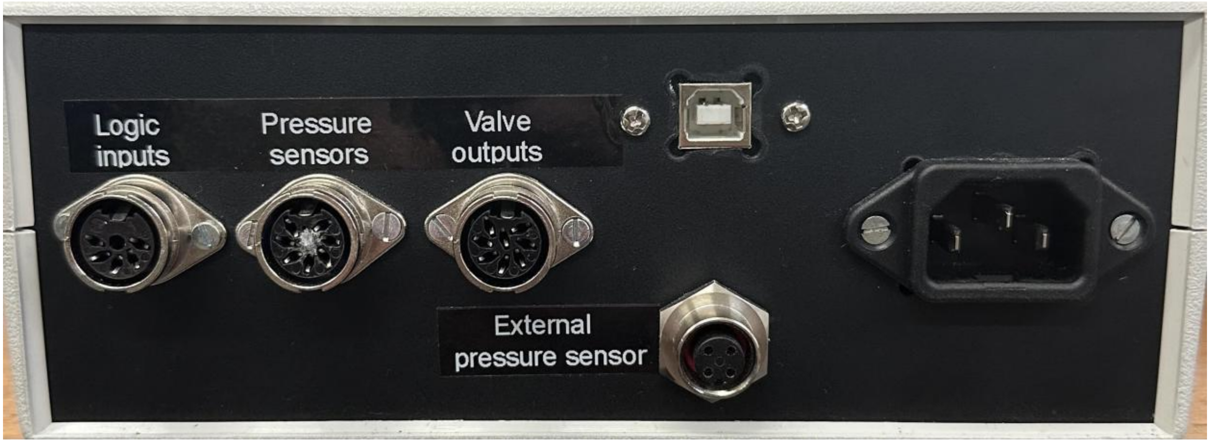

Figure S10: Rear view photograph of the electronics enclosure for the solenoid valve manifold.

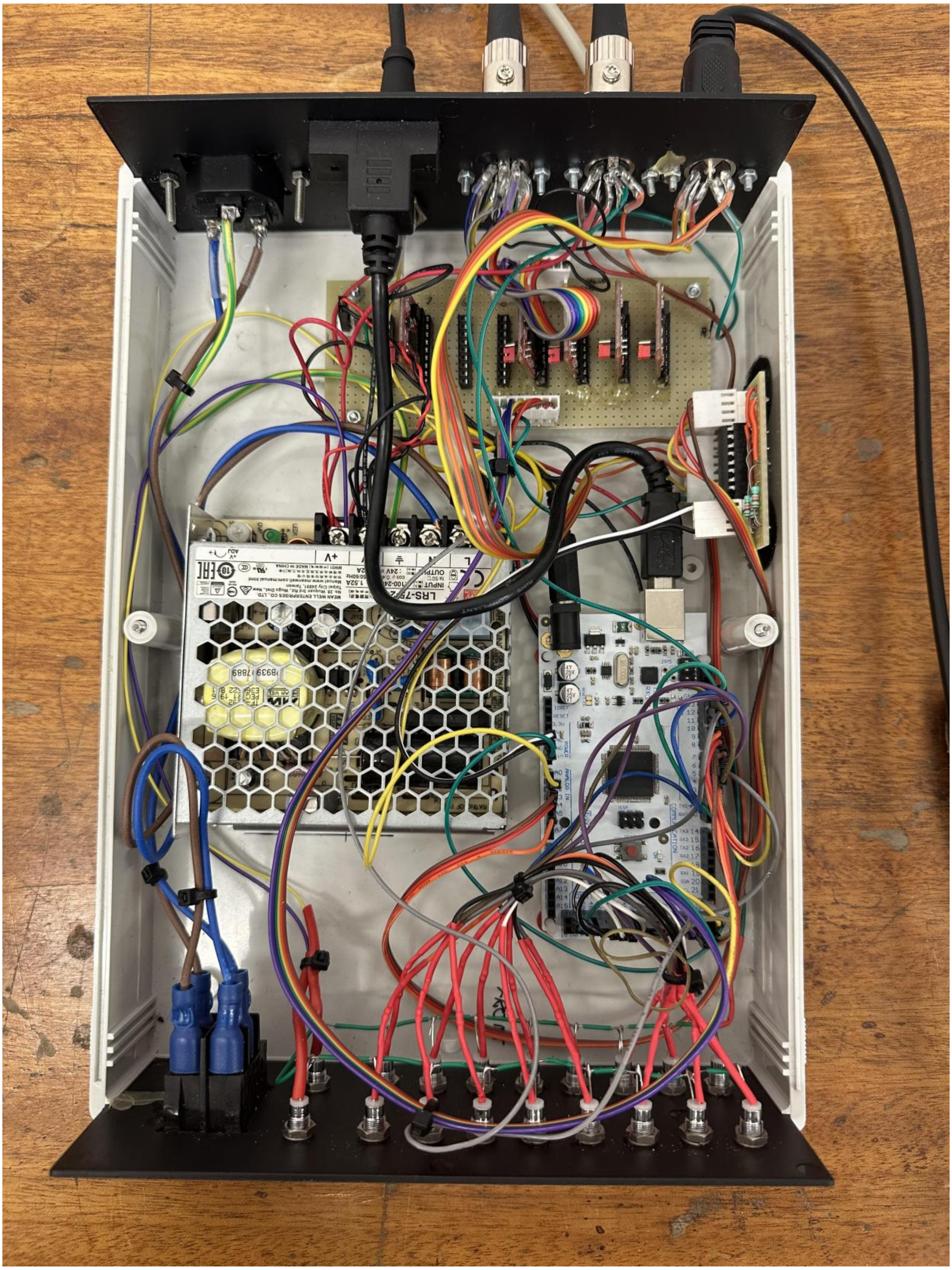

Figure S11: Photograph of the electronic components used to control the solenoid valve manifold.

Table S3: Bill of materials for the electronic components of the solenoid valve manifold.

Manufacturer	Product code	Quantity	Unit price / £ (excl. VAT)	Vendor	Function/Description
Hammond Manufacturing	1598JSGYPBK	1	11.71	Rapid Electronics	Plastic enclosure
Kemo Electronic	E005	1	3.26	Onecall	Stripboard
Mean Well	LRS-35-24	1	10.07	Rapid Electronics	230 V AC/DC 24 V power supply
Multicomp Pro	MP004803	1	2.10	Onecall	Power switch
Multicomp Pro	MC19020001	8	1.24	Onecall	Green LEDs indicating valve switch status
Multicomp Pro	MC19020002	8	1.53	Onecall	Yellow LEDs indicating valve power status
Multicomp Pro	MC19020003	2	1.27	Onecall	Red LEDs indicating 24 V and 9V status
Multicomp Pro	2MT3000-W04300	1	6.45	Onecall	M12 socket
NResearch	CDS-V01	5	15.56	NResearch	CoolDrive Solo valve driver cards
PrehKeyTec	71206-051/0801	1	2.90	Onecall	5-pin DIN socket
PrehKeyTec	71206-070/0800	1	4.43	Onecall	7-pin DIN socket
PrehKeyTec	71206-080/0800	1	4.15	Onecall	8-pin DIN socket
Pro Signal	PSG-JWS-65	1	1.84	Onecall	Jumper wire set
Schurter	6100.3200	1	0.60	Onecall	C14 socket
StarTech	USBPNLBFBM1	1	4.49	Onecall	USB B socket
Traco Power	TSR 1-2490	1	7.20	Rapid Electronics	24/9 V DC/DC converter
Whadda	WPB101	1	23.41	Onecall	ATmega2560 development board
Wurth Elektronik	61300911821	8	4.46 (10/PK)	RS Components	Board-to-board receptacle for mounting valve driver cards

S4. Custom NMR tube and gas adaptor

The custom NMR tube consists of a standard 5 mm O.D. NMR tube fused to a GL14 thread for a total length of 255 ± 0.2 mm.

The custom gas adaptor comprises two parts: an acetal co-polymer main body, and a polytetrafluoroethylene (PTFE) adaptor that interfaces with the NMR tube.

The main body is a 27 mm diameter, 35 mm long cylinder, machined with two full-length, diametrically opposed locating flats to a width of 26 mm. Each flat features a 1/4-28 UNF threaded hole, centred 19 mm from the top face (1.5 mm vertical offset), to secure the adaptor within the linear actuator's 3D-printed sample holder (**Section S5**). The top face houses central and 8 mm offset 1/4-28 UNF threaded ports (accepting IDEX® Super Flangeless™ fittings). The central port connects to a 3.3 mm concentric through-hole, which accommodates a 1/16" O.D. capillary that delivers gas directly into the NMR sample. Return gas flows up the annular space surrounding this capillary, and is routed to the offset port *via* a perpendicular cross-drill; the manufacturing access for this channel is spotfaced (17 mm diameter, 1.5 mm depth) and sealed with a G 1/8 nitrile blanking plug and bonded seal. The bottom face contains an R 1/8 threaded port, into which the PTFE adaptor is screwed.

The PTFE adaptor is machined from 15 mm rod with a 2.5 mm concentric bore running its full length (34.5 mm). A 12.5 mm diameter, 2 mm thick flange separates the component into two sections: an upper segment (length 20.5 mm, diameter 10 mm) containing an R 1/8 male thread (length 15 mm) that screws into the main body, and a lower segment (length 12 mm) machined to an 8.5 mm diameter cylinder that inserts into the NMR tube. To secure the assembly, a bored-through GL14 screw cap slides over the adaptor's upper section and screws onto the NMR tube, compressing the glass rim against the flange to form a face seal.

Figures S12 and **S13** provide dimensioned technical drawings of the components of the custom gas adaptor. **Table S4** provides a parts list for the custom NMR tube and gas adaptor.

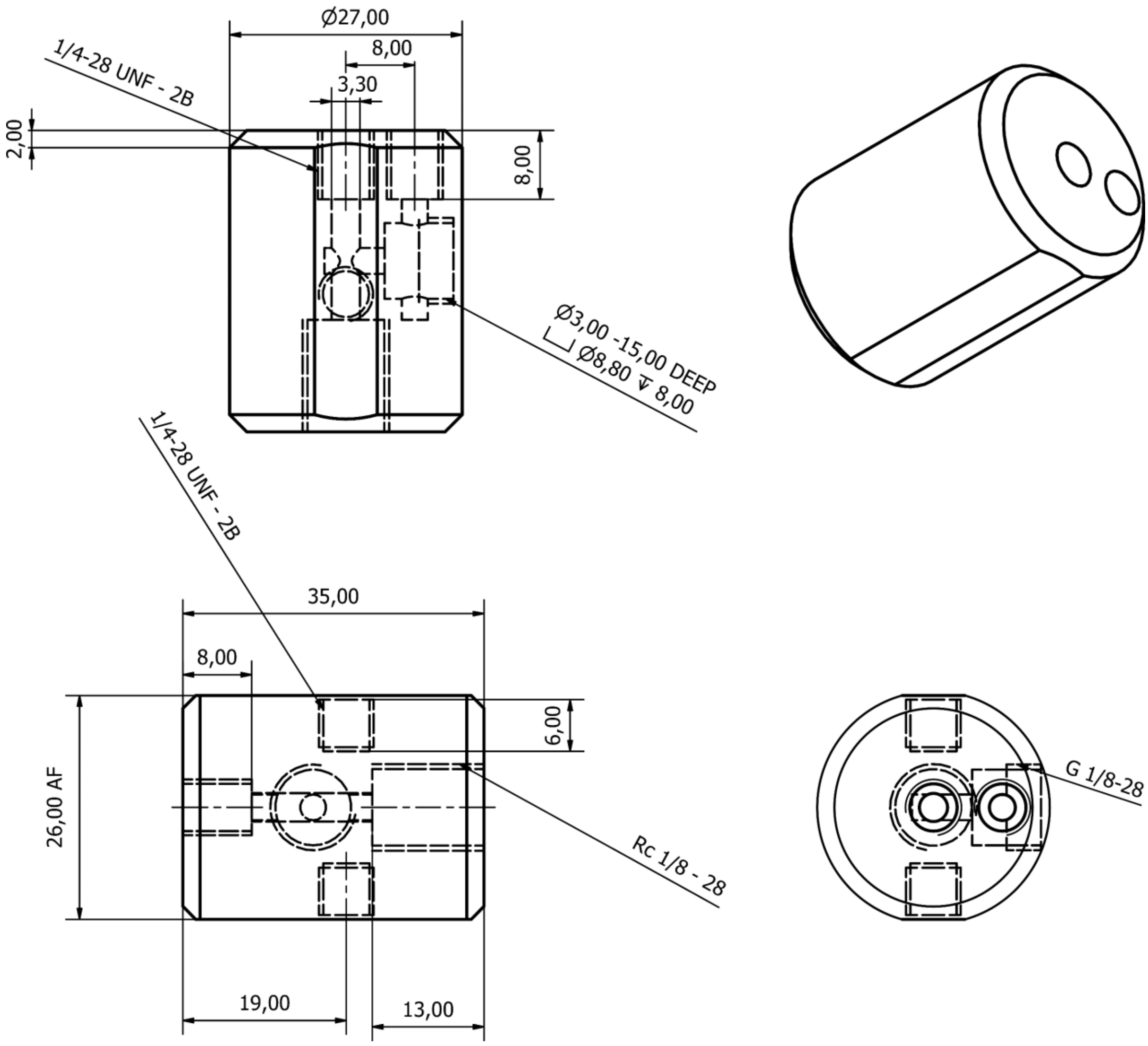

Figure S12: Technical drawing of the acetal co-polymer main body component of the custom gas adaptor.

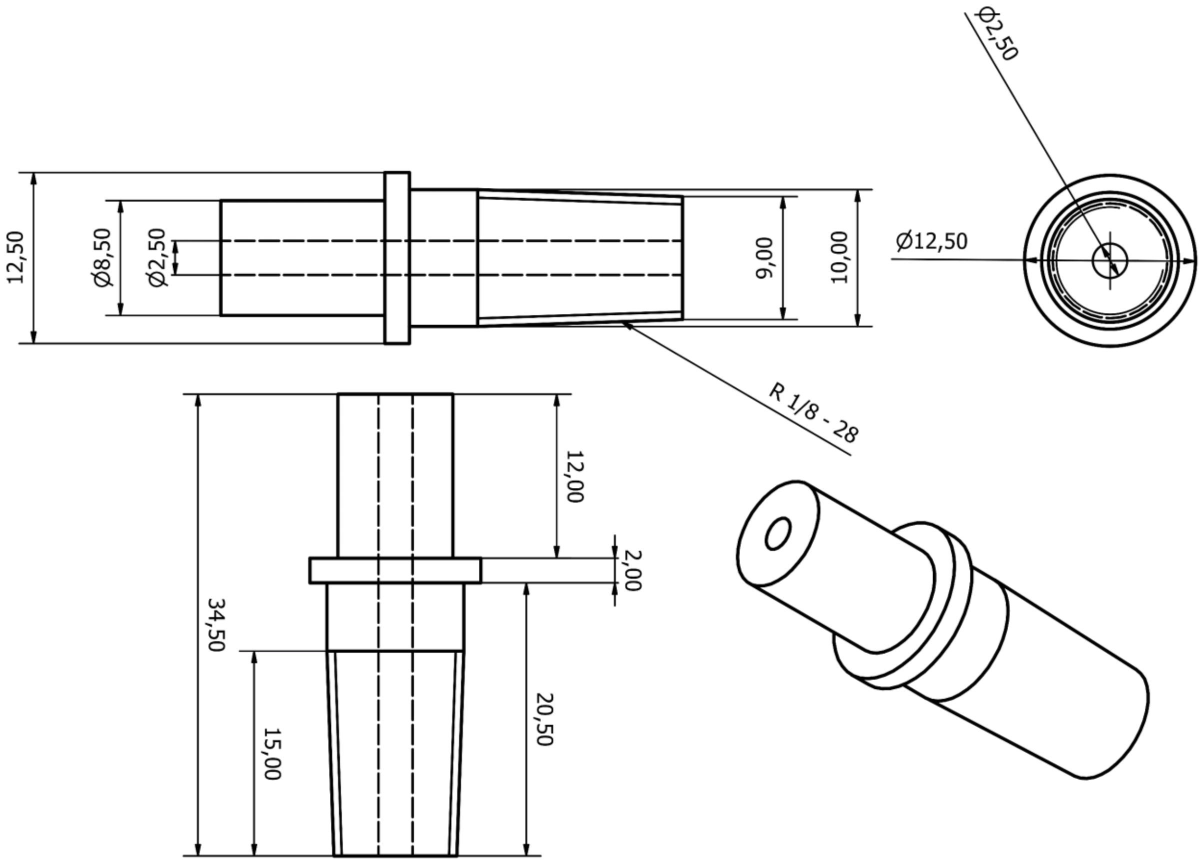

Figure S13: Technical drawing of the PTFE NMR tube adaptor component of the custom gas adaptor.

Table S4: Bill of materials for the custom NMR tube and gas adaptor.

Manufacturer	Product code	Quantity	Unit price / £ (excl. VAT)	Vendor	Function/Description
A Plus	AP-668-01	1	3.57	Dixon Science	GL14 cap, bored-through
Direct Plastics	ACNAR02805	1	5.77	Direct Plastics	Acetal rod, 28 mm diameter
Direct Plastics	PTR01505	1	8.88	Direct Plastics	PTFE rod, 15 mm diameter
Duran	248360207	1	31.20 (10/PK)	VWR	GL14 screw thread tube, attachable
Eastern Seals	BON-820-4490-41(2.00)	1	19.40 (100/PK)	Eastern Seals	1/8” BSP nitrile bonded seal
RS Pro	176-1048	1	8.75 (10/PK)	RS Components	G 1/8 nitrile blanking plug
Wilmad	528-PP-7	1	14.40	Apollo Scientific	5 mm O.D. NMR tube

S5. Linear actuator

The linear actuator consists of an ACME 8 mm lead screw coupled to a single gantry plate through an anti-backlash nut block, providing 400 mm of travel along a 500 mm 40 × 80 mm (C-Beam) linear rail. A NEMA23 hybrid bipolar stepper motor (2.40 N·m holding torque) synchronously drives the lead screw 1:1, achieving a constant linear velocity of 260 $mm \cdot s^{-1}$ and an acceleration of 930 $mm \cdot s^{-2}$. The actuator interfaces with the custom NMR tube and gas adaptor (**Section S4**) using an aluminium bracket bolted to the gantry plate, to which a 3D-printed polylactic acid sample holder is attached. Two 1/4-28 UNF screws securely hold the gas adaptor in the holder, ensuring the sample tube remains parallel to the bore of the NMR spectrometer whilst in motion. A machined aluminium base plate mounts the actuator on top of a Magritek Spinsolve benchtop NMR spectrometer using three M5 screws, which precisely align the sample tube concentrically with the bore. The design for the base plate was initially optimised using 3D printing. The stereolithography file for 3D printing is included in the data repository. The actuator is supported by a frame constructed using 20 × 20 mm and 20 × 40 mm aluminium extrusion, which rests on top of the NMR spectrometer. **Figure S14** provides a photograph of the system. **Table S5** provides a parts list for replication of the system.

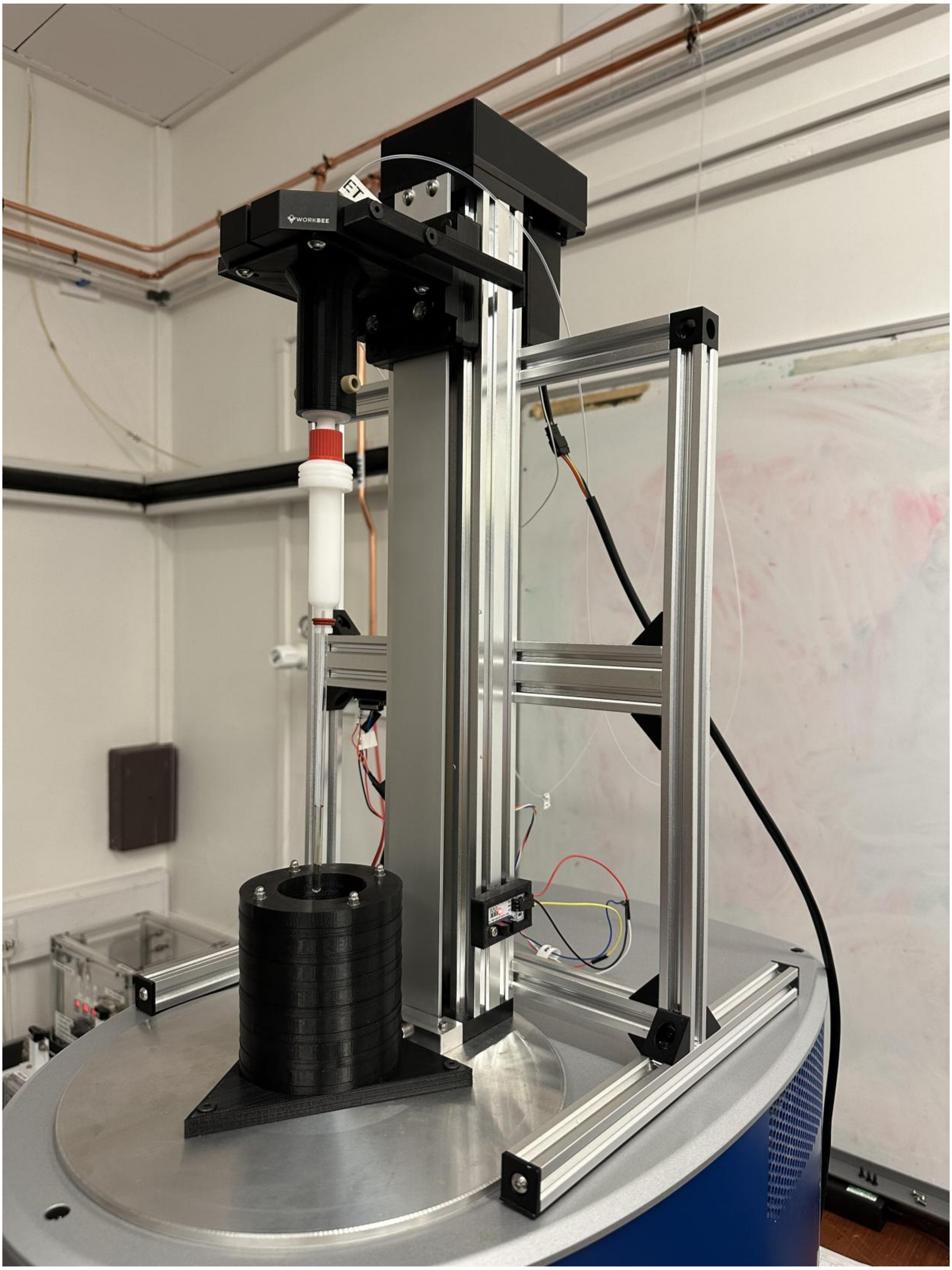

Figure S14: Photograph of the linear actuator mounted on top of a Magritek Spinsolve benchtop NMR spectrometer.

Table S5: Bill of materials for replication of the linear actuator.

Manufacturer	Product code	Quantity	Unit price / £ (excl. VAT)	Vendor	Function/Description
Ideal Power	15DYS902-320300G	1	33.50	Onecall	AC/DC power supply for uStepper S32 controller
Ooznest	CBEAM-LA-CP	1	188.92	Ooznest	C-Beam linear actuator:<br>Length: 500 mm<br>Colour: silver anodise<br>Gantry plate: single gantry<br>Motor: 24.5 kg·cm (345 oz·in) 3.00 A 1.8º NEMA23 hybrid bipolar stepper motor<br>Shield: C-Beam linear actuator shield kit<br>Motor standoff: C-Beam linear actuator motor standoff kit<br>Controller: uStepper S32 controller
Ooznest	DCA-PTA	1	2.46	Ooznest	DC socket adaptor for uStepper S32 controller
Ooznest	WB-Z1P-M-RM-43-K	1	41.25	Ooznest	Aluminium bracket for interfacing custom NMR tube and gas adaptor to linear actuator
Ooznest	ESTOP-O	2	2.08	Ooznest	Optical endstop
Ooznest	AEP-TV-2020-S-CTS	2	3.85	Ooznest	20 x 20 mm aluminium extrusion for supporting frame<br>Length: 390 mm<br>Angle cutting: no angles<br>M5 tapping: both ends tapped

Ooznest	AEP-TV-2020-S-CTS	2	3.12	Ooznest	20 x 20 mm aluminium extrusion for supporting frame<br>Length: 290 mm<br>Angle cutting: no angles<br>M5 tapping: both ends tapped
Ooznest	AEP-TV-2020-S-CTS	2	3.19	Ooznest	20 x 20 mm aluminium extrusion for supporting frame<br>Length: 300 mm<br>Angle cutting: no angles<br>M5 tapping: both ends tapped
Ooznest	AEP-TV-2040-S-CTS	1	5.51	Ooznest	20 x 40 mm aluminium extrusion for supporting frame<br>Length: 290 mm<br>Angle cutting: no angles<br>M5 tapping: both ends tapped
Ooznest	CCB-20-3	2	3.25	Ooznest	20 x 20 mm three-way cube corner brackets for connecting aluminium extrusion components of frame
Ooznest	CB-20-2020-C	12	0.95	Ooznest	20 x 20 mm 90° corner brackets for connecting aluminium extrusion components of frame
Ooznest	AEC-20-2020-F	4	2.38	Ooznest	20 x 20 mm end caps for frame
Ooznest	DIN-20-M5	34	0.18	Ooznest	M5 drop in nuts for connecting between and mounting to aluminium extrusion components
Volex	X-299960A	1	4.56	Onecall	Mains power cord for uStepper S32 controller

S6. Polarisation transfer field

A cylindrical Halbach array, mounted on top of the NMR spectrometer, provides the 6.39 ± 0.14 mT B_{PTF} used in all experiments. To accommodate sample shuttling, the cylinder's inner diameter was designed to be larger than those typically used in manual SABRE experiments.[1] The array geometry was optimised using analytical magnetic field modelling tools from the *Magpylib* Python library. The final design consists of seven 3D-printed polylactic acid (PLA) discs, each containing four equidistant 5 × 5 × 5 mm N42 neodymium magnets (First4Magnets®, product code F5CU) at a radius of 32 mm. The magnet centres are vertically separated by 13.5 mm using 8.5 mm 3D-printed PLA spacer discs, forming a 94.5 mm long cylinder with an outer diameter of 90 mm and an inner diameter of 50 mm. A 9.5 mm thick base secures the Halbach array on the NMR spectrometer, ensuring concentricity between the bore of the spectrometer, Halbach array, and sample tube. **Figure S15** provides dimensions for the magnet and spacer discs of the Halbach array. **Figure S16** shows the magnetic field strength across the length of the array.

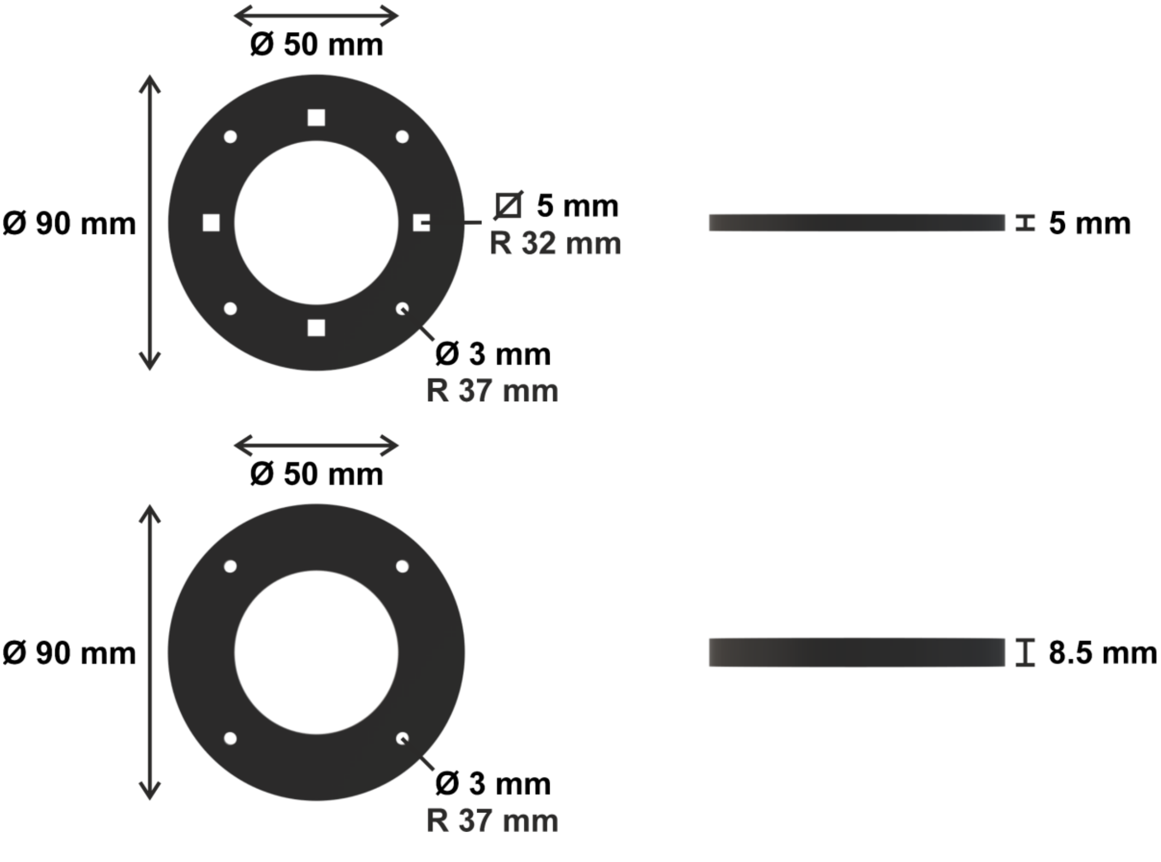

Figure S15: Dimensioned renders of the magnet (top) and spacer (bottom) discs used in the construction of the 6.39 ± 0.14 mT Halbach array.

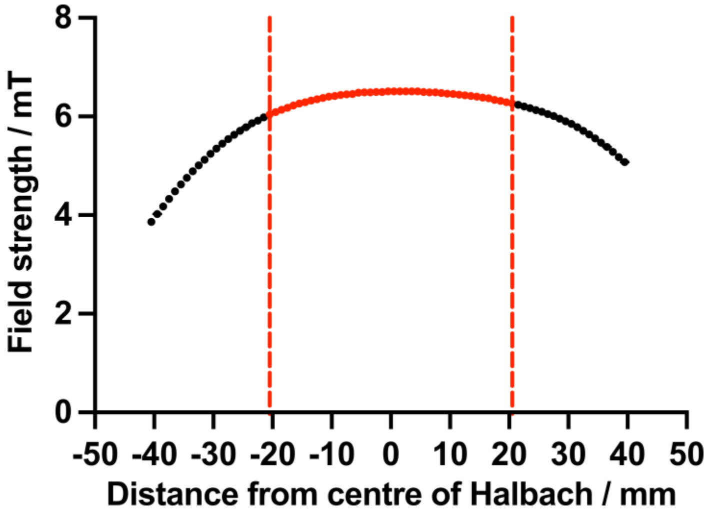

Figure S16: Transverse magnetic field strength as a function of distance along the z-axis from the centre of the Halbach array. The magnetic field was measured in 1 mm increments between the centres of the top and bottom magnet discs ($\Delta z = 81$ mm). Data plotted are the mean of three measurements. The magnetic field is deemed to be uniform over a distance ± 20.5 mm (red data points), with an average field strength of 6.39 ± 0.14 mT.

S7. SABRE-enhanced $^{13}C\{^{1}H\}$ benchtop NMR SNR comparison

The signal-to-noise ratio (SNR) values for the SABRE-enhanced $^{13}C\{^{1}H\}$ benchtop NMR spectra from this work (**Fig. 5b**), and from the works of Robinson *et al.*[2] and Kircher *et al.*,[3] are presented in **Table S6**. The corresponding experimental parameters are summarised in **Table S7**.

Table S6: SABRE-enhanced $^{13}C\{^{1}H\}$ signal-to-noise ratio values.

		**This work**	**Robinson *et al.*[2]**	**Kircher *et al.*[3]**
**4-methylpyridine**	*ortho*	17	31	
	meta	19	23	
	para	26	91	
	methyl	16	73	
**pyridine**	*ortho*	10		
	meta	10		
	para	10		
**3.5-dimethylpyridine**	*ortho*	9		
	meta	40		
	para	18		
	methyl	33		
**Average**	*ortho*	12		
	meta	23		
**4-aminopyridine**	*ortho*			143
	meta			66
	para			n/a

Table S7: Summary of experimental parameters.

	**This work**	**Robinson *et al.*[2]**	**Kircher *et al.*[3]**
**Concentration / mM**	3	260	60
**Number of scans (*N*)**	64	1	1
**pH_2 / %**	51	99	99
**Detection field / T**	1.4	1	1.4

Due to the large number of differences in the experimental parameters and the different substrates used, we can only make a rough comparison of the SNR values with previous results in the literature. The reduction in *para*hydrogen enrichment from *ca.* 100 % to *ca.* 50 % is expected to decrease the polarisation and hence the SNR by a factor of 3,[4] while signal averaging (N = 64 *vs*. N = 1) is expected to increase the SNR by a factor of 8. Therefore, an efficiency ratio can be estimated for our current work relative to the previously reported results (A / B) using the following equation.

$$\text{Ratio} = \frac{SNR}{SNR_{\text{A/B}}} \times \frac{[\text{A/B}]}{3\text{ mM}} \times \frac{3}{8}$$

The efficiency ratios are reported in **Table S8**. There is a clear increase in efficiency relative to the results of Robinson *et al.*,[2] with ratios ranging from 7 to 27. However, there will be a small SNR benefit in our work because we are using a 1.4 T detetion field (compared to 1 T). Using the average SNR for the *ortho* and *meta* peaks of each of the three components of our mixture for the comparison with the work of Kircher *et. al*., the efficiency ratios are 0.6 and 2.6 for the *ortho* and *meta* peaks, respectively. Given the large number of approximations made in this calculation, this comparison is not definitive. However, it suggests that the overall level of hyperpolarisation efficiency is comparable in the two methods, with better efficiency achieved by the *in situ* method for the *ortho* carbon, while the indirect transfer method achieves higher efficiency across the molecule. This is in line with expectations, given the reliance of the direct polarisation method on the coupling between the hydride ligands on the SABRE catalyst and the target carbon on the bound substrate.

Table S8: Effiency ratio between this work and previously.

	**Robinson *et al.*[2]**	**Kircher *et al.*[3]**
ortho	27	0.6
meta	9	2.6
para	18	
methyl	7	

S8. Single-component spectra

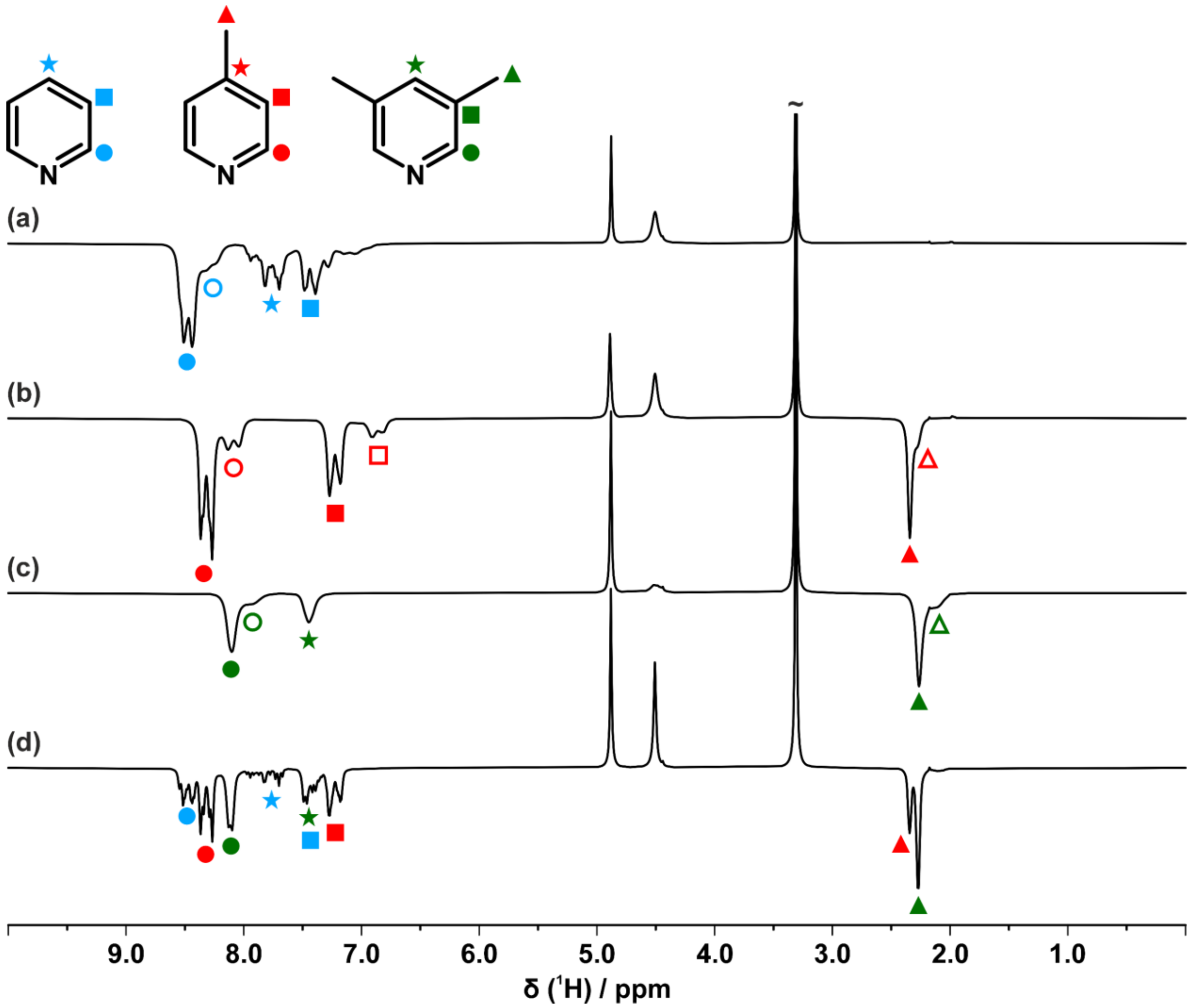

Figure S17: (a)-(c) SABRE-enhanced benchtop ^{1}H NMR spectra of **(a)** pyridine, **(b)** 4-methylpyridine, and **(c)** 3,5-dimethylpyridine single-component samples. Each sample contained 25 mM of the target molecule and 5 mM [IrCl(COD)(IMes)] SABRE pre-catalyst in methanol. Single-component spectra show enhanced peaks for each target molecule in free solution (solid symbols) and bound to the active SABRE catalyst (open symbols). The combination of free and bound peaks increases the issue of signal overlap in **(d)** the SABRE-enhanced benchtop ^{1}H NMR spectrum of a mixture of these three components (3 mM each). Peaks at δ(^{1}H) = 3.34 and 4.90 ppm correspond to solvent C<u>H</u>$_3$ and O<u>H</u>, respectively. The peak at δ(^{1}H) = 4.50 ppm corresponds to hyperpolarised *ortho*hydrogen in solution.

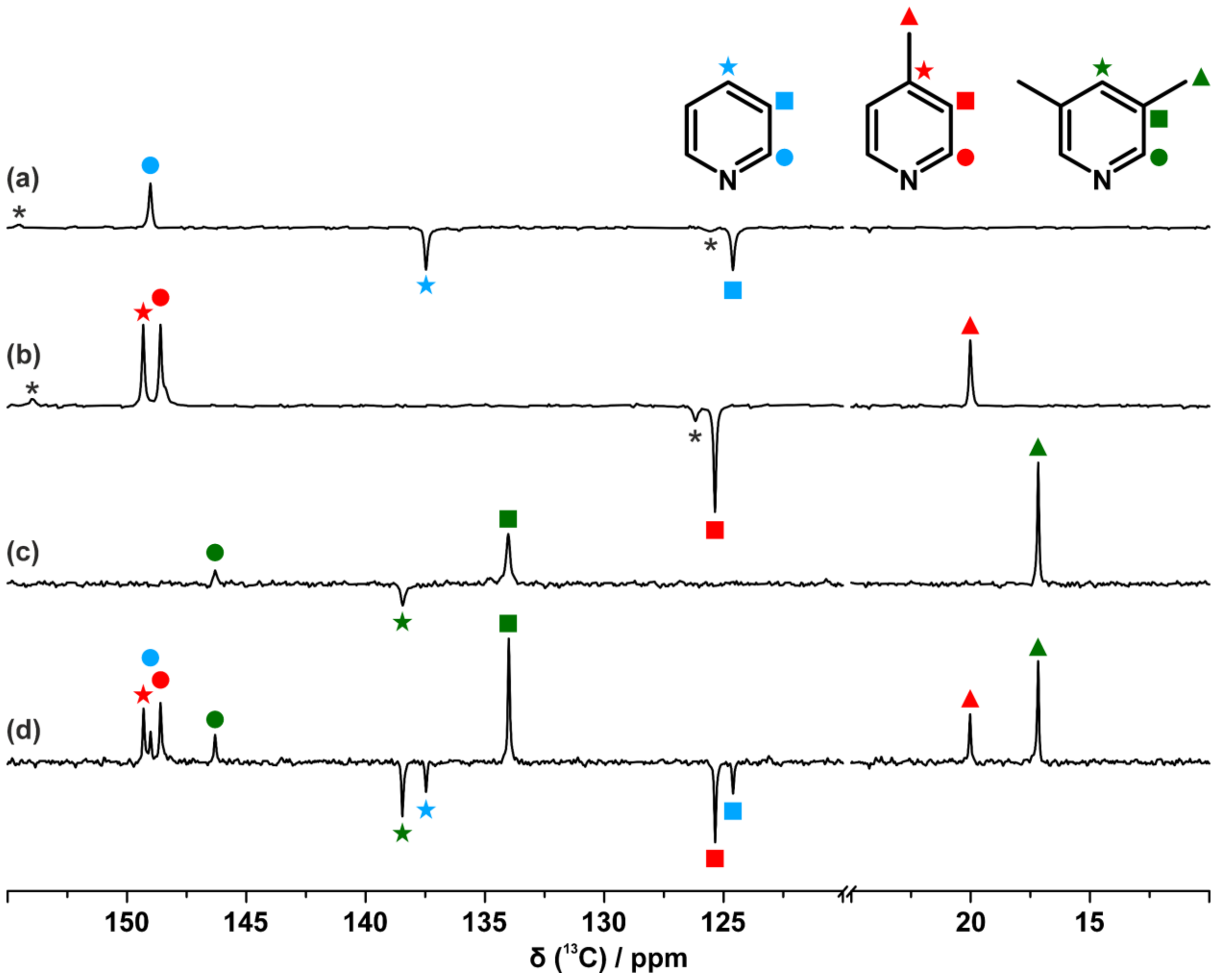

Figure S18: (a)-(c) SABRE-enhanced benchtop $^{13}C\{^1H\}$ INEPT NMR spectra of **(a)** pyridine, **(b)** 4-methylpyridine, and **(c)** 3,5-dimethylpyridine single-component samples. Each sample contained 25 mM of the target molecule and 5 mM [IrCl(COD)(IMes)] SABRE pre-catalyst in methanol. Peaks in **(a)** and **(b)** marked with an asterisk likely correspond to the $^{13}C\{^1H\}$ signals of pyridine and 4-methylpyridine bound to the active SABRE catalyst, respectively. **(d)** SABRE-enhanced benchtop $^{13}C\{^1H\}$ INEPT NMR spectrum of the mixture of these three components (3 mM each).

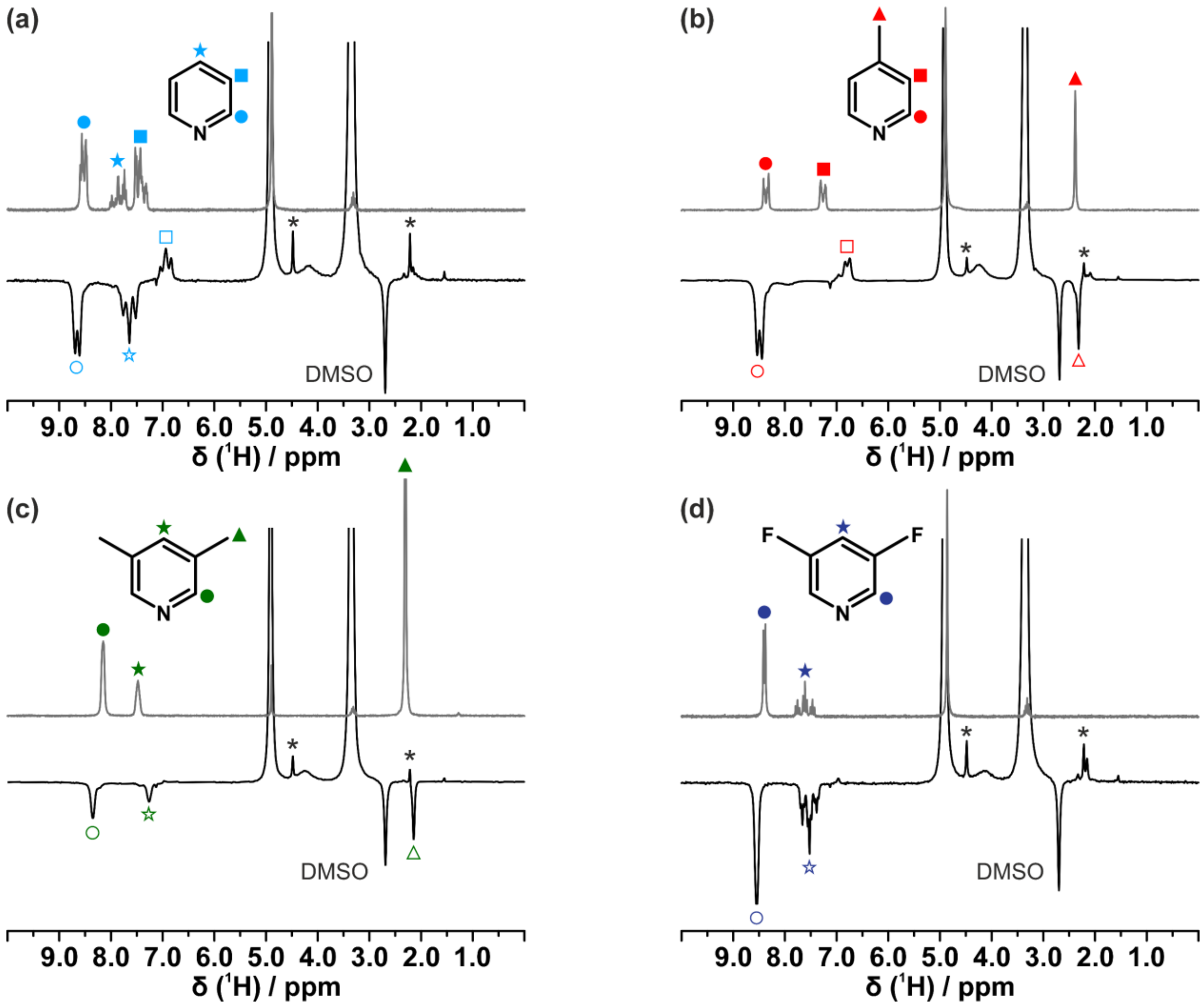

Figure S19: Comparison of SABRE-enhanced (black; 500 µM substrate, 5 mM [IrCl(COD)(IMes)] SABRE pre-catalyst, 25 mM DMSO in methanol) and thermal reference (grey; 250 mM substrate in methanol-*d*4) ^{1}H benchtop NMR spectra for **(a)** pyridine, **(b)** 4-methylpyridine, **(c)** 3,5-dimethylpyridine, and **(d)** 3,5-difluoropyridine. In all cases, the SABRE-enhanced peaks appear shifted relative to the reference sample in the absence of the catalyst. This suggests that the observed hyperpolarisation in this concentration range, and in the presence of DMSO, is dominated by molecules bound to the SABRE catalyst, and not those in free solution. This is consistent with previous observations for 3,5-difluoropyridine with DMSO as a co-substrate.[5] The peak at δ(^{1}H) = 4.50 ppm corresponds to hyperpolarised *ortho*hydrogen in solution. Peaks marked with an asterisk correspond to methanol ^{13}C satellites. Polarisation transfer was carried out in the fringe field of the benchtop NMR spectrometer (inside the bore). The inhomogeneity of B_{PTF} leads to the patterns of positive and negative enhancements observed for pyridine and 4-methylpyridine.

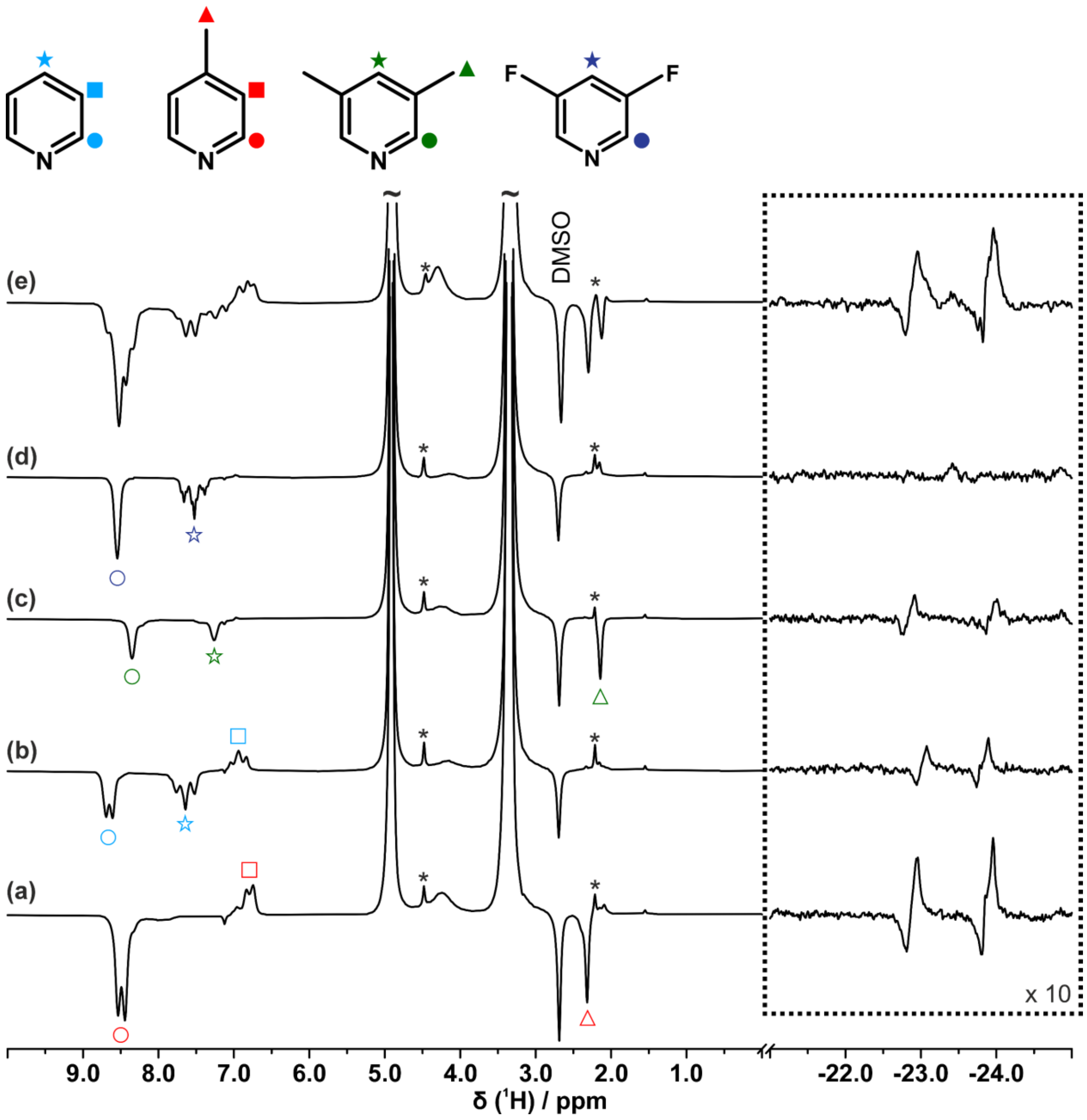

Figure S20: (a)-(d) SABRE-enhanced benchtop ^{1}H NMR spectra of **(a)** 4-methylpyridine, **(b)** pyridine, **(c)** 3,5-dimethylpyridine, and **(d)** 3,5-difluoropyridine single-component samples. Each sample contained 500 μM of the target molecule, 5 mM [IrCl(COD)(IMes)] SABRE pre-catalyst, and 25 mM DMSO in methanol. **(e)** SABRE-enhanced benchtop ^{1}H NMR spectrum of the mixture of these four components (750 μM each). The hydride region (dashed box) is vertically scaled 10-fold to show the form of the hyperpolarised hydride resonances in each spectrum. Polarisation transfer was carried out in the fringe field of the benchtop NMR spectrometer (inside the bore). The inhomogeneity of B_{PTF} leads to the patterns of positive and negative enhancements observed for pyridine and 4-methylpyridine.

S9. Repository contents

The following files generated as part of this research are openly available from the research data repository of the University of York (DOI: 10.15124/d941c05a-ab67-4da1-8382-6bd0eb3ae39d).

1. Raw NMR data for **Figs. 4–7** and **Figs. S17–S20**.
2. Stereolithography (STL) files for 3D-printing the linear actuator's sample holder, base plate, optical endstop mounting brackets and endstop triggers (**Section S5**), and the discs used in the Halbach array (**Section S6**).
3. Source codes uploaded to Whadda ATmega2560 and uStepper S32 microcontrollers for PC control of valve manifold and linear actuator, respectively.
4. Macros to control valve manifold and linear actuator from within the SpinsolveExpert environment.
5. Macros for the SpinsolveExpert graphical user interfaces for manual control of the valve manifold and linear actuator.
6. Example pulse program and experiment control macro for integration of valve manifold/linear actuator control into an NMR experiment.

S10. References

1 P. M. Richardson, S. Jackson, A. J. Parrott, A. Nordon, S. B. Duckett and M. E. Halse, *Magn. Reson. Chem.*, 2018, **56**, 641–650.

2 A. D. Robinson, P. M. Richardson and M. E. Halse, *Appl. Sci.*, 2019, **9**, 1173.

3 R. Kircher, J. Xu and D. A. Barskiy, *J. Am. Chem. Soc.*, 2024, **146**, 514–520.

4 P. M. Richardson, R. O. John, A. J. Parrott, P. J. Rayner, W. Iali, A. Nordon, M. E. Halse and S. B. Duckett, *Phys. Chem. Chem. Phys.*, 2018, **20**, 26362–26371.

5 A. I. Silva Terra and M. E. Halse, *Chemistry–Methods*, 2025, **5**, e202400094.
